# Legacy effects of continuous chloropicrin-fumigation for 3-years on soil microbial community composition and metabolic activity

**DOI:** 10.1186/s13568-017-0475-1

**Published:** 2017-09-18

**Authors:** Shuting Zhang, Xiaojiao Liu, Qipeng Jiang, Guihua Shen, Wei Ding

**Affiliations:** 1 Laboratory of Natural Products Pesticides, College of Plant Protection, Southwest University, No.2 Tiansheng Road, Beibei, Chongqing, 400715 China; 2 Department of Microbial Ecology, Netherlands Institute of Ecology, NIOO-KNAW, Wageningen, Netherlands

**Keywords:** Continuous fumigation, Bacterial community structure, *Actinobacteria*, *Saccharibacteria*

## Abstract

Chloropicrin is widely used to control ginger wilt in China, which have an enormous impact on soil microbial diversity. However, little is known on the possible legacy effects on soil microbial community composition with continuous fumigation over different years. In this report, we used high throughput Illumina sequencing and Biolog ECO microplates to determine the bacterial community and microbial metabolic activity in ginger harvest fields of non-fumigation (NF), chloropicrin-fumigation for 1 year (F_1) and continuous chloropicrin-fumigation for 3 years (F_3). The results showed that microbial richness and diversity in F_3 were the lowest, while the metabolic activity had no significant difference. With the increase of fumigation years, the incidence of bacterial wilt was decreased, the relative abundance of *Actinobacteria* and *Saccharibacteria* were gradually increased. Using LEfSe analyses, we found that *Saccharibacteria* was the most prominent biomarker in F_3. Eight genera associated with antibiotic production in F_3 were screened out, of which seven belonged to *Actinobacteria*, and one belonged to *Bacteroidetes.* The study indicated that with the increase of fumigation years, soil antibacterial capacity may be increased (possible reason for reduced the incidence of bacterial wilt), and *Saccharibacteria* played a potential role in evaluating the biological effects of continuous fumigation.

**Electronic supplementary material:**

The online version of this article (doi:10.1186/s13568-017-0475-1) contains supplementary material, which is available to authorized users.

## Introduction

Soil fumigants are used extensively to control soil-borne pests including nematodes, pathogens, and weeds, so that increasing the yields of many crops (Mao et al. 2013; Wang et al. 2014; Ibekwe 2004). For instance, bacterial wilt on ginger, caused by the soil-borne bacterial pathogen *Ralstonia solanacearum*, is a major disease responsible for enormous yield losses (Yang et al. 2012). Without soil fumigation, it can cause an 80% crop failure in ginger (Li et al. 2014). Therefore, in order to ensure a better crop yield and provide greater benefits to farmers, soil fumigation has become an important global agricultural practice (Ajwa et al. 2010). Methyl bromide (MB) is mainly used for pre-planting fumigation of vegetables to manage many kinds of phytopathogenic organisms including *R. solanacearum* (Paret et al. 2010). However, MB had been phased out in developing countries owing to its detrimental effects on the stratospheric ozone layer (Bell et al. 2000). Chloropicrin is a potential replacement for MB, which has been widely used to control ginger bacterial wilt in China (Mao et al. 2013).

Soil bacteria have a high proportion of microorganisms, and they are responsible for many beneficially biological functions in biogeochemical and nutrient cycles, disease suppression, organic matter formation and decomposition, soil structure and plant growth promotion (Chaparro et al. 2012; Kennedy 1999). In addition, soil bacteria play an important role in soil-borne disease suppression (She et al. 2016). Rhizosphere bacteria are antagonistic to soil-borne pathogens by preventing their infection on hosts (Latz et al. 2012). It is possible to bring back the soil suppressive status in a way changing the composition of rhizosphere bacteria (van Agtmaal et al. 2015).

Fumigation can greatly change the bacterial composition and decrease the abundance and diversity of bacterial community (Chen et al. 2016). However, the *Actinomycetes* and gram-positive bacteria have been reported to recover preferentially after 90 days of fumigation (Klose et al. 2006). Liu et al. demonstrated that 1,3-D had a rapid but short-lived impact on the indigenous soil bacteria community (Liu et al. 2015). Chloropicrin significantly altered bacterial population within 4 weeks of application, and the effect on bacterial population structure became non-significant 4 months later after treatment (Wei et al. 2016). So recent studies showed that the decrease of microbiome after fumigation could be mostly short-term, while the reconstructed system can exist for a long period (Yao et al. 2006). Therefore, it’s necessary to compare the legacy effects of fumigation at a single time point, especially after crops planted. In addition, it is not clear what happens to the soil bacterial community after several years of continuous fumigation.

According to our survey, we found that the incidence of ginger wilt was approximately 5% in continuous fumigation for 3 years by chloropicrin; however, in non-fumigated fields, the value was approximately 40%. Therefore, we hypothesized that the dominant bacteria in the soil can be screened out by continuous fumigation, which may give a positive effect on ginger bacterial wilt. We analyzed the changes of soil bacteria structure diversity of non-fumigation (NF), chloropicrin-fumigation for 1 year (F_1) and continuous chloropicrin-fumigation for 3 years (F_3). Specifically, this study aimed to evaluate the biological legacy effects of continuous fumigation. The results will deepen our understanding of the possible bacterial mechanisms underlying continuous fumigation to control bacterial wilt.

## Materials and methods

### Soil sampling

**Fig. 1:**
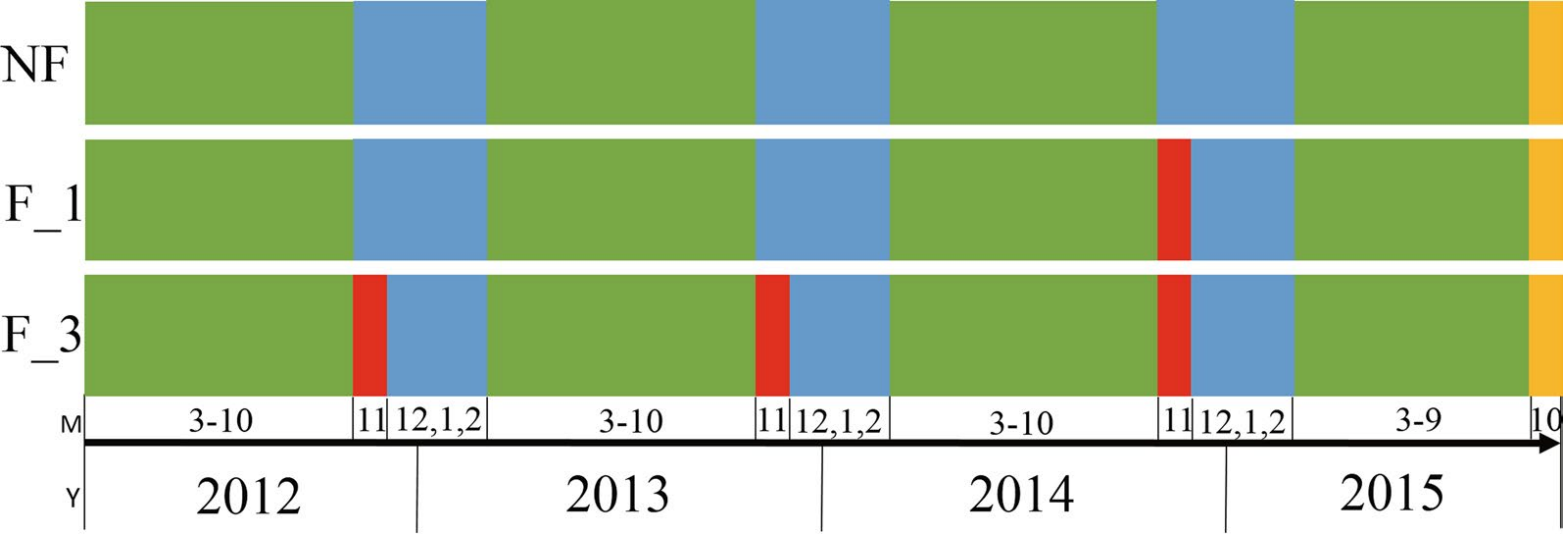
The sample processing information. The green represents ginger growth period. The blue represents fallow period. The red represents fumigated treatment. The yellow represents sample collection period. *M* month, *Y* year, *NF* non-fumigation, *F_1* chloropicrin-fumigation for 1 year, *F_3* continuous chloropicrin-fumigation for 3 years

The samples were collected to study different fumigation years on ginger wilt soil microbiota in Weifang city, Shandong Province, China. The samples were collected from soils that non-fumigated (NF), fumigated for 1 year (F_1), and continuously fumigated for 3 years (F_3). The F_1 was fumigated in November 2014, and the F_3 was fumigated every November from 2012 to 2014. The ginger’s growth period was from March to October every year. The details relevant to these trials are given in Fig. 1. The fumigant was chloropicrin (Dalian Dyestuffs & Chemicals Co., China), a commercial liquid product containing 99.5% chloropicrin. Chloropicrin liquid was injected into the soil at a 15 cm depth via a manual injection machine at a rate of 50 g/m^2^. After fumigating, the soil was mulched by 0.04 mm impermeable film for 15 days. We uniformly collected samples in October 2015 during the early ginger harvest. The distance between each sampling point was within 100 m, and the ecological environment was consistent. Each treatment had three replicates, of which was collected and mixed to a composite sample from five randomly point. The soil cores were sampled at the 5–20 cm soil depth. After removal of the plant residues, the samples were transported to the laboratory in an ice chest (4 °C). The basic physical and chemical properties of each soil sample is in Additional file 1: Table S1.

### DNA extraction and sequencing library construction

Total genomic DNA was extracted from 0.4 g of soil using an Omega Biotek Soil DNA Kit (Omega Biotek, USA), following the standard protocol. DNA concentration and purity was checked on 1% (w/v) agarose gels. PCR amplifications were conducted with primers 338 forward (5′-ACTCCTACGGGAGGCAGCAG-3′) and 806 reverse (5′-GGACTACHVGGGTWTCTAAT-3′), which amplified the V3–V4 region of the 16S rDNA gene (Xu et al. 2016).

Polymerase chain reaction amplification consisted of an initial denaturation at 95 °C for 3 min, 28 cycles of denaturation at 95 °C for 30 s, annealing at 55 °C for 30 s, elongation at 72 °C for 45 s, and a final extension at 72 °C for 10 min. PCR reactions were performed in triplicate using 20 µL reactions with 2.0 µL 10× buffer, 2.0 µL dNTP (2.5 mM), 0.2 µL rTaq Polymerase (Takara), 0.2 µL BSA, 0.8 µL each of forward and reverse primers, and 10 ng of template. Amplified products were run on 2% agarose gel for identification; samples with bright main strips between 400 and 450 bp were chosen for further experiments. Amplicons were combined at roughly equal amplification intensity ratios, purified using an AxyPrep DNA Gel Recovery Kit (AXYGEN) and submitted to the next-generation sequencing laboratory at Majorbio Bio-pharm Technology Co.,Ltd., Shanghai, China for Illumina paired-end library preparation, cluster generation, and 250-bp paired-end sequencing. The raw reads have been deposited into the NCBI short-reads archive database under Accession Number SRP096942.

### Microbial metabolic activity

To explore the microbial metabolic activity profiles, Biolog ECO microplates (EcoPlate™, BIOLOG, Hayward, CA, USA) were employed (Zhang et al. 2014). The plate (96-well) contained 31 sole carbon sources and a blank (control well with water), of which the 31 different carbon sources can be grouped into six kinds of carbon sources, including carbohydrates, amino acids, polymers, amines, phenolic acids and carboxylic acids, and each plate was reproduced in triplicate. Weighing 5 g fresh samples (dry weight), each was mixed with 45 mL of sterile 0.85% NaCl solution in 100 mL Erlenmeyer flasks and then shook for 30 min at 170 r/min. After standing for 20 min, the suspension was diluted 150-fold with sterile water. 150 μL of diluted suspensions were inoculated into each well of the ECO microplates, which were then incubated at 28 °C for 168 h. Color development was measured with a ThermoFisher SCIENTIFIC (MULTISKAN GO, USA) microplate reader at 590 nm every 24 h after inoculation.

The AWCD can directly reflect the overall carbon source metabolic activity of microbes (Garland and Mills 1991). AWCD_590nm_ was used as an indicator of general microbial activity and was assessed as the average optical density of all wells per plate (Zhang et al. 2014). The AWCD was calculated as described by Wang et al. (2016).

### Statistical analysis

Raw Illumina fastq files were demultiplexed, quality filtered, and analyzed using QIIME v1.7.0 (Quantitative Insights Into Microbial Ecology) (Caporaso et al. 2010). Operational taxonomic units (OTUs) were picked with a threshold of 97% pairwise identity. High-confidence OTUs were identified using the following criteria: any sample had a collective abundance of greater than 20 reads. Subsampling by the minimum number of sample sequences. A comparison of overall microbial distribution in different fumigation years was conducted on the relative abundances of phyla, which were calculated using OTUs based on taxonomy, using Origin 9.0 software for histogram analysis.

We calculated the Chao and Shannon indexes as measures of α-diversity (within-sample species richness). For β-diversity analysis, dissimilarity of bacterial communities was determined using cluster analysis based on Bray–Curtis dissimilarity and principal coordinate analysis (PCoA) on unweighted and weighted UniFrac distances among all samples. Linear discriminant analysis (LDA) effect size (LEfSe) employed the factorial Kruskal–Wallis sum-rank test (α = 0.05) to identify taxa with significant differential abundances between categories (using one-against-all comparisons), followed by LDA to estimate the effect size of each differentially abundant feature (logarithmic LDA score >2.0). Significant taxa were used to generate taxonomic cladograms, which illustrated the differences between sample classes on the website http://huttenhower.sph.harvard.edu/galaxy. Additionally, the taxonomic levels were limited from domain to genus in case of the distraction from redundant data.

Mean and standard error for each set of data were calculated by one-way analysis of variance (ANOVA) with Turkey’s honest significant difference test (P < 0.05) using SPSS software (version 17.0).

## Results

### 16S rRNA high-throughput sequencing results of bacterial community composition

#### Overall distribution

**Fig. 2:**
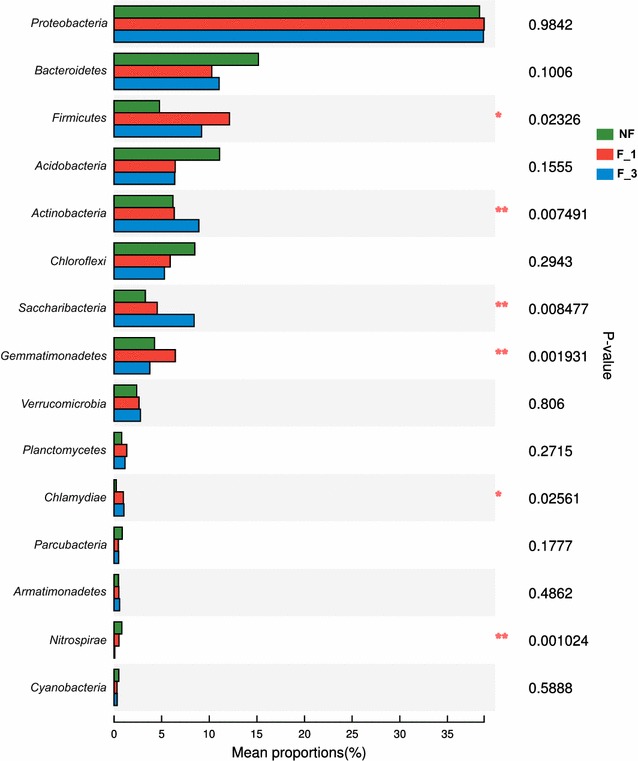
Average relative abundance of the mainly abundant phyla within the different fumigation years (n = 3). Stars indicate significant enrichment (One-way ANOVA, P < 0.05; Multiple testing adjustment, FDR) in the different fumigation years. 0.01 < P ≤ 0.05, one star; 0.001 < P ≤ 0.01, two stars; P ≤ 0.001, three stars. *NF* non-fumigation, *F_1* chloropicrin-fumigation for 1 year, *F_3* continuous chloropicrin-fumigation for 3 years

**Fig. 3:**
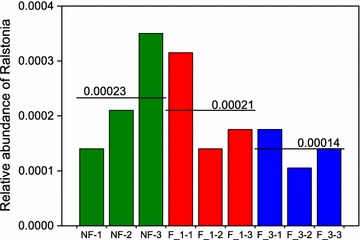
The relative abundance of genus *Ralstonia* in different fumigation years. The solid line represents the average. *NF* non-fumigation, *F_1* chloropicrin-fumigation for 1 year, *F_3* continuous chloropicrin-fumigation for 3 years

A total of 28,557 reads and 3433 bacterial operational taxonomic units (OTUs) were obtained from the nine samples through 16S rRNA high-throughput sequencing analysis. The average read length was 438 bp. These bacterial OTUs were assigned to 38 different phyla. The F_3 library included the maximum number of phyla (37), and the F_1 library had the minimum (35). *Proteobacteria* was the dominant phyla in all soils, followed by *Bacteroidetes* (Fig. 2). When compared F_3 with the NF, *Actinobacteria* and *Saccharibacteria* were significantly increased by 3 and 5%, respectively (P < 0.05; Additional file 1: Table S2). In addition, the relative abundance of phyla *Saccharibacteria* and *Actinobacteria* were increased as the fumigation years increased. However, *Nitrospirae* was opposite. The incidence of bacterial wilt was significantly reduced by fumigation, and the F_3 had the lowest incidence (Additional file 1: Figure S1). And the genus *Ralstonia*, the main taxon of ginger bacterial wilt, was reduced as the fumigation years increased (Fig. 3), but not significantly.

#### Alpha diversity

**Fig. 4:**
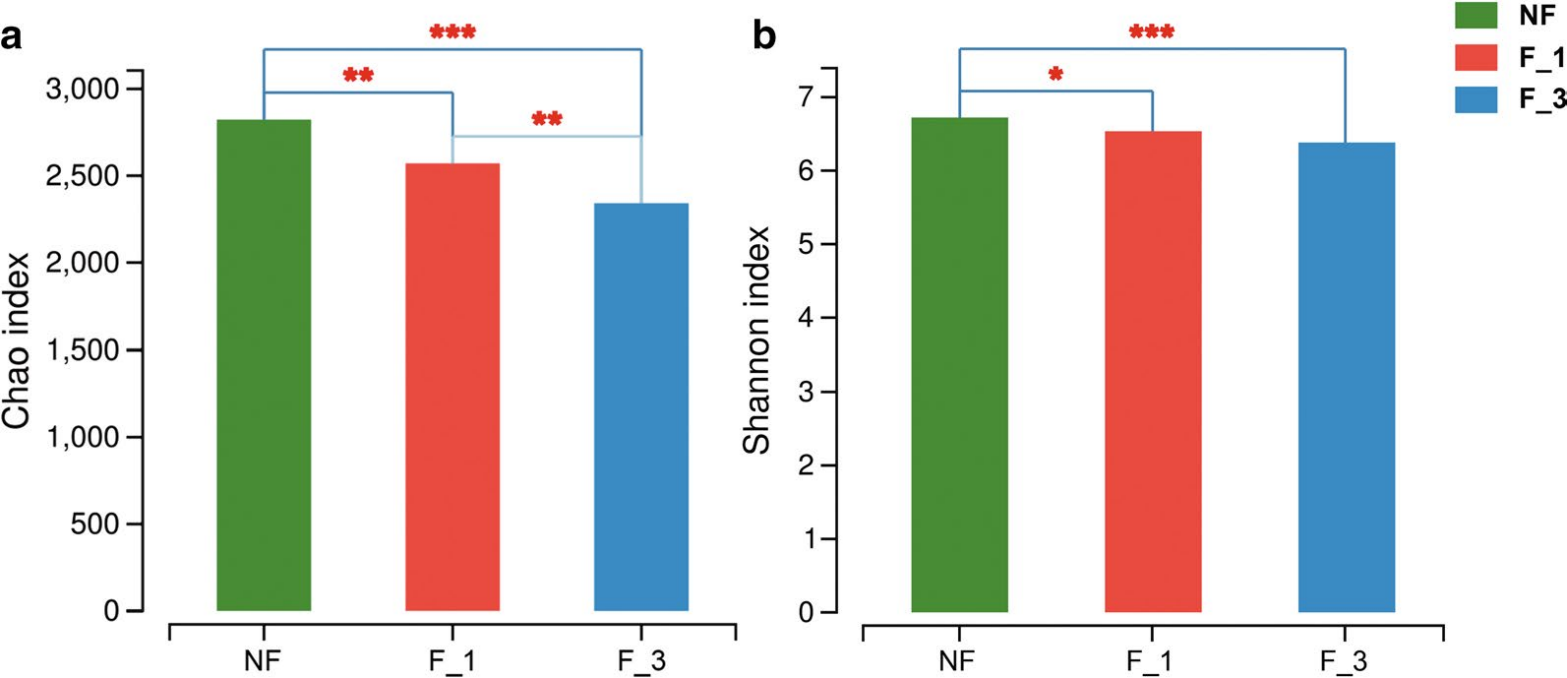
Microbiota richness **a** and diversity **b** as measured by Chao and Shannon indexes observed in the different fumigation years. P values was from two-tailed Student’s *t* test (P < 0.05). 0.01 < P ≤ 0.05, one star; 0.001 < P ≤ 0.01, two stars; P ≤ 0.001, three stars. *NF* non-fumigation, *F_1* chloropicrin-fumigation for 1 year, *F_3* continuous chloropicrin-fumigation for 3 years

The rarefaction curves are illustrated in Additional file 1: Figure S2. This rarefaction curve indicated a large variation in the total number of OTUs in the different samples, but the sequence coverage was still sufficient to capture the diversity of the bacterial communities. The F_3 had the lowest richness index of Chao (*P* = 2×10^−3^, *P* = 1×10^−4^ and *P* = 6×10^−3^, Student’s *t* test; NF-F_1, NF-F_3 and F_1-F_3, respectively). Figure 4a and the Shannon diversity index was significantly reduced compared to NF (*P* = 7×10^−4^ and *P* = 0.059, Student’s *t* test; NF-F_1 and NF-F_3, Fig. 4b). With the increasing time of fumigation, both richness and diversity saw significant reductions.

#### Beta diversity

**Fig. 5:**
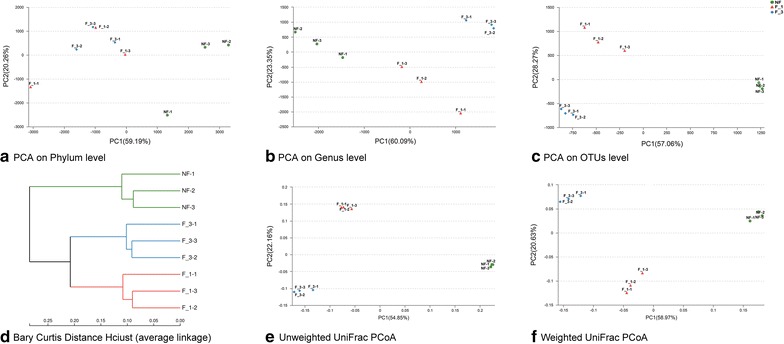
The beta diversity of fumigated soil samples in different years. **a** PCA based on phylum level; **b** PCA based on genus level; **c** PCA based on total OTUs; **d** Cluster analysis based on bray–curtis. **e** PCoA analysis with unweighted UniFrac; **f** PCoA analysis with weighted UniFrac. *NF* non-fumigation, *F_1* chloropicrin-fumigation for 1 year, *F_3* continuous chloropicrin-fumigation for 3 years

Different beta diversity statistical analyses were used to identify the relationships among different fumigation years. The results of principal component analysis (PCA) between phyla (Fig. 5a) and genus (Fig. 5b) levels were different. At phylum level, the F_1 and F_3 were difficultly separated, but a much better separation was exhibited at the genus level. The results indicated that these communities shared similar phyla diversity. As shown by cluster analysis (Fig. 5d), the samples from the same fumigation years were grouped tightly, and the samples from the fumigated and non-fumigated fields could be separated into two lineages. The profiles of the bacterial community structure were plotted using PCA based on OTU (Fig. 5c) and PCoA based on unweighted (Fig. 5e) and weighted UniFrac (Fig. 5f) distances between all samples. The results indicated that the fumigation samples shared a high similarity rate in their bacterial structure. The PCA, clustering and PCoA results obviously showed that the bacterial communities between fumigated and non-fumigated fields have high differentiation.

#### The discriminative taxa analyzed by LEfSe

**Fig. 6:**
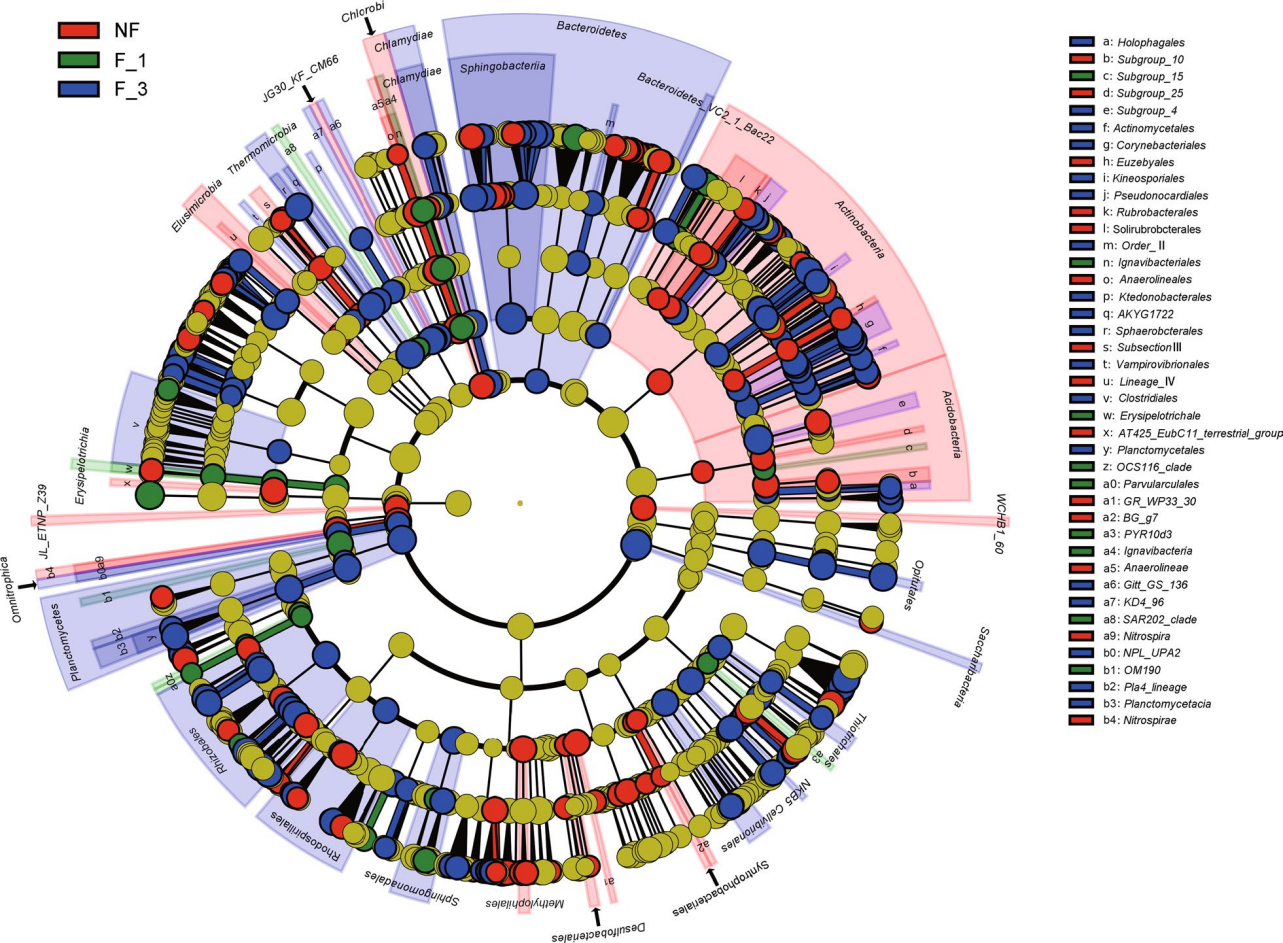
Lefse cladogram of the aggregated group of NF, F_1, and F_3. A range of bacterial taxa from phylum to genus level was associated with the NF (red), F_1 (green) and with F_3 (blue) (α = 0.05, LDA > 2.0, the size of circles is proportionate to each taxon’s mean relative abundance). The yellow circles represents no significantly different taxa. *NF* non-fumigation, *F_1* chloropicrin-fumigation for 1 year, *F_3* continuous chloropicrin-fumigation for 3 years

Least discriminant analysis effect size (LEfSE) was used to identify the key phylotypes responsible for the differences among NF, F_1 and F_3 (Fig. 6). There were 276 taxa distinguished three groups: 106 for NF, 28 for F_1, and 142 for F_3 (Additional file 1: Date S1). *Nitrospirae* and *Saccharibacteria* were the most prominent phylum in NF and F_3, respectively. There was no biomarker selected out in F_1 at the phylum level. At the genus level, NF, F_1, and F_3 screened out 52, 12 and 69 major taxa, respectively. In these taxa, the F_3 had 18 taxa belonging to *Actinobacteria,* but F_1 only had one, and NF had eight (Table 1).

**Table 1:** The screen out genera by LEfSe belong to phylum *Actinobacteria*

Sample	Order	Family	Genus	LDA score (log10)
NF	*Micrococcales*	*Intrasporangiaceae*	*Ornithinimicrobium*	4.21
NF	*Rubrobacterales*	*Rubrobacteriaceae*	*Rubrobacter*	4.71
NF	*Acidimicrobiales*	*Acidimicrobiaceae*	*Illumatobacter*	5.91
NF	*Propionibacteriales*	*Nocardioidaceae*	*Aeromicrobium*	6.26
NF	*Euzebyales*	*Euzebyaceae*	*Euzebya*	4.70
NF	*Micromonosporales*	*Micromonosporaceae*	*Dactylosporangium*	4.82
NF	*Micromonosporales*	*Micromonosporaceae*	*Rhizocola*	4.35
NF	*Gaiellales*	*Gaiellaceae*	*Gaiella*	6.05
F_1	*Streptosporangiales*	*Nocardiopsaceae*	*Nocardiopsis*	5.18
F_3	*Corynebacteriales*	*Corynebacteriaceae*	*Corynebacterium_1*	6.96
F_3	*Micrococcales*	*Brevibacteriaceae*	*Brevibacterium*	6.46
F_3	*Streptosporangiales*	*Streptosporangiaceae*	*Nonomuraea*	6.33
F_3	*Micrococcales*	*Microbacteriaceae*	*Leucobacter*	6.40
F_3	*Corynebacteriales*	*Mycobacteriaceae*	*Mycobacterium*	6.09
F_3	*Streptosporangiales*	*Thermomonosporaceae*	*Actinomadura*	5.93
F_3	*Micromonosporales*	*Micromonosporaceae*	*Luedemannella*	5.85
F_3	*Pseudonocardiales*	*Pseudonocardiaceae*	*Pseudonocardia*	5.80
F_3	*Corynebacteriales*	*Dietziaceae*	*Dietzia*	5.72
F_3	*Acidimicrobiales*	*Acidimicrobiales_Incertae_Sedis*	*Aciditerrimonas*	5.56
F_3	*Corynebacteriales*	*Nocardiaceae*	*Rhodococcus*	5.65
F_3	*Kineosporiales*	*Kineosporiaceae*	*Angustibacter*	5.48
F_3	*Frankiales*	*Frankiaceae*	*Jatrophihabitans*	4.39
F_3	*Pseudonocardiales*	*Pseudonocardiaceae*	*Amycolatopsis*	5.33
F_3	*Propionibacteriales*	*Nocardioidaceae*	*Kribbella*	4.87
F_3	*Actinomycetales*	*Actinomycetaceae*	*Flaviflexus*	5.15
F_3	*Micrococcales*	*Bogoriellaceae*	*Bogoriella*	5.19
F_3	*Pseudonocardiales*	*Pseudonocardiaceae*	*Actinophytocola*	4.77

#### Microbial metabolic activity

**Fig. 7:**
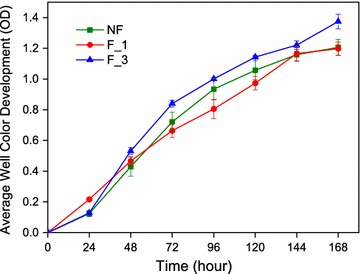
Kinetics of the average well color development (AWCD_590 nm_) curve of bacterial communities of different fumigation years (mean ± SE, n = 3). *NF* non-fumigation, *F_1* chloropicrin-fumigation for 1 year, *F_3* continuous chloropicrin-fumigation for 3 years

**Fig. 8:**
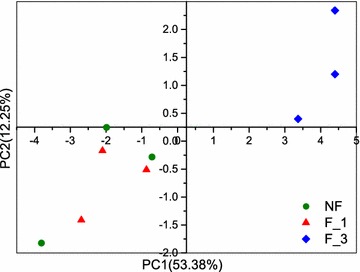
Principal component analysis (PCA) of carbon utilization of the soil microbial community (72 h). *NF* non-fumigation, *F_1* chloropicrin-fumigation for 1 year, *F_3* continuous chloropicrin-fumigation for 3 years

The average well color development (AWCD) was typically conducted to measure the total microbial metabolic activity by reflecting carbon source utilization ability. The AWCD of NF was higher than F_1 during the incubation period of 72–120 h and became undifferentiated after 144 h. The F_3 had the highest AWCD after incubation at 48 h (Fig. 7). However, the microbial metabolic activity exhibited no significance difference in all samples (P < 0.05). A principal component analysis (PCA) in culture for 72 h was performed (Fig. 8). There was no significant difference in NF and F_1 between carbon source utilization. However, the F_3 had a significant difference in carbon source utilization compared to NF and F_1. In summary, after 3 years of continuous fumigation, the use of the carbon source was significantly changed, but the metabolic activity was not significantly enhanced.

## Discussion

Soil fumigation can significantly impact the soil microbial communities (Ibekwe et al. 2001). Fumigants can reduce the bacterial population in the early stage and create a temporary “biological vacuum”. The temporary vacant niche can be easily and quickly recolonized by many bacteria (Yakabe et al. 2010). The effects on the bacterial population structure are short-lived and become non-significant difference compared to non-fumigation after fumigating 2–3 months (Liu et al. 2015; Wei et al. 2016). In this study, with the increase of fumigation years, the richness and diversity were significantly reduced. Although the soil microbial population structure could be restored after fumigation, the microbial richness and diversity can not be restored to the original state.

To investigate whether the pattern of reduction in microbial composition would apply to metabolic activity, we analyzed the microbial catabolic diversity of fumigated field under different years. However, the result indicated that although the microbial richness and diversity in F_3 were the lowest, its metabolic activity had no significant difference compared to NF and F_1. The species with the same or similar functions in the community can functionally substituted for each other, and the species, whose function can be completely replaced by other species, were regarded as a functional redundancy of the community (De 2017; Cowling 1994; Rosenfeld 2002). To some extent, the Biolog EcoPlate method can be used to assess the functional diversity of microbial communities by examining the amount of different C metabolizable substrates (Nannipieri et al. 2003). Due to the functional redundancy of microbiological taxa, function-similar taxa compete and supplement, fumigation can significantly change the microbial diversity, but can not affect the metabolic activity of microorganisms.

Fumigants play an important role in control of soil-borne diseases. The combination use of chloropicrin and 1,3-dicloropropene provided a good control of *Fusarium* wilt in tomato and cucumber (Yan et al. 2017). And drip-applied chloropicrin efficiently managed *Verticillium* wilt in pepper (Ślusarski and Spotti 2016). Duo to the certain antibacterial property, plant essential oils, could act as a biofumigant and provided an effective control of bacterial wilt in ginger (Paret et al. 2010). Soil biofumigation with brassica plant residues can prevent the development of pepper *Phytophthora* blight caused by *Phytophthora capsici* through altering soil microbial community structures (Wang et al. 2014). Chloropicrin can sharply reduce the population of *R. solanacarum* in highly infested soil (Mao et al. 2013). Whether chloropicrin can control the bacterial wilt by changing soil microbial community structures.

*Actinobacteria* is known as the decomposer of organic materials, such as cellulose and chitin, and thereby plays an important role in organic matter turnover and C cycling (Sykes and Skinner 1973). Meanwhile, *Actinobacteria* possess the ability to produce a wealth of important natural products, especially antibiotics, which suppress the growth and development of a wide range of soil dwelling plant pathogens (Garbeva et al. 2004; Janvier et al. 2007; Luo et al. 2014). Previous studies showed that 140–160 antibiotics have been used in human therapy and agriculture, of which 100–120 were produced by *Actinobacteria* (Stach et al. 2003b). The relative abundance of *Actinobacteria* in F_1 was increased by 0.11% compared to NF, but was not significantly. However, F_3 was significantly increased by 2.80 and 2.69% compared to NF and F_1, respectively. These indicated that the continuous fumigation increased the abundance of *Actinobacteria* (Additional file 1: Table S2; Fig. 2).

Using LEfSE we found 18 genera belonging to *Actinobacteria* in F_3 (Additional file 1: Date S1; Table 1). Claverías et al. (2015) screened out 17 genera belonging to the phylum *Actinobacteria,* which showed antimicrobial activity, and 4 genera, *Dietzia*, *Flaviflexus*, *Pseudonocardia*, and *Rhodococcus*, were found in this study. In addition, the genera *Amycolatopsis* (Dávila Costa and Amoroso 2014; Singh et al. 2012), *Actinomadura* (Dirlam et al. 1990), and *Pseudonocardia* (Cafaro and Currie 2011) originally have attracted researchers’ attention duo to their antibiotic producing capabilities. Notably, the order *Actinomycetales,* which was only screened out in F_3. Some studies have shown that the order *Actinomycetales* represents the most prominent group of microorganisms for the production of bioactive compounds, especially antibiotics and antitumor agents (Goodfellow and Fiedler 2010; Stach et al. 2003a).

*Chitinophaga,* a genus belonging to phylum *Bacteroidetes,* is one of the biomarkers in F_3 (Additional file 1: Date S1) and was first described by Sangkhobol and Skerman (1981) when including strains of filamentous, chitinolytic, and gliding bacteria that are transformed into spherical bodies during aging. Previously, studies showed that *Chitinophaga* was the only known isolated *Bacteroidetes* antibiotic producer (Wyatt et al. 2013). Elansolid A was isolated as the first macrolide antibiotic from *Chitinophaga sancti* (Steinmetz et al. 2011). Remarkably, Elansolid A occurred as two stable and separable atropisomers, A1 (1) and A2 (1*) (Jansen et al. 2011). In this study, continuous fumigation altered the soil microbial community structures in two ways: (1) reducing the relative abundance of *Ralstonia*; and (2) increasing the number of bacteria that could produce antibiotics. Therefore, these may be the reason why continuous fumigation for 3 years significantly reduced the incidence of ginger bacterial wilt (Additional file 1: Figure S1).

The development of genome sequencing technology enhances our understanding of the microbial world; however, since most microorganisms are not culturable, and the existing databases can not completely name the microorganism (Rinke et al. 2013). In this study, the abundance of phylum *Saccharibacteria* increased due to the increase of chloropicrin-fumigated years (Fig. 2) and *Saccharibacteria* was the most prominent biomarker (Log LDA score = 7.22) in F_3 (Additional file 1: Date S1). The phylum candidatus *Saccharibacteria* was formerly known as Candidate Division TM7. It is a well-known and ubiquitous bacterial phylum in soils, sediments, wastewater and animals, as well as in a host of clinical environments (Ferrari et al. 2014). However, due to its non-culturable stage, how the main function of *Saccharibacteria* is involved in potential disease control remains unknown. Only if the functions of *Saccharibacteria* were clear, the effects of continuous fumigation on the soil microbial community structure would be more explicit. After continuous fumigation for 11 months had been finished, there was still a clear legacy effect on bacterial community composition with a strong increase in relative abundance of the phyla *Actinobacteria* and *Saccharibacteria*. And soil fumigation has a certain screening effect on soil microbes, *Actinobacteria* and *Saccharibacteria* gradually dominated in soil microbes by continuous fumigation, which played an important role in controlling ginger bacterial wilt.

## Additional file

**Additional file 1: Figure S1.** Disease incidence of ginger bacterial wilt. **Figure S2.** The rarefaction curve of samples. **Table S1.** Soil Physicochemical Data. **Table S2.** The top ten Phyla of samples. **Dataset S1.** Discriminative taxa analyzed by LEfSe in all samples.

## Abbreviations

NF: non-fumigation
F_1: chloropicrin-fumigation for 1 year
F_3: continuous chloropicrin-fumigation for 3 years
DNA: deoxyribonucleic acid
rRNA: ribosomal ribonucleic acid
PCR: polymerase chain reaction
ECO: ecoplate
AWCD: average well color development
OUT: operational taxonomic units
PCA: principal component analysis
PCoA: principal coordinates analysis
LEfSE: least discriminant analysis effect size
LDA: linear discriminant analysis
FDR: falsely discovery rate
