# Supplementary material for: Legacy effects of continuous chloropicrin-fumigation for 3-years on soil microbial community composition and metabolic activity
Source: AMB Express. 2017 Sep 18;7:178. doi: 10.1186/s13568-017-0475-1 (PMC5603465; doi:10.1186/s13568-017-0475-1)
Supplement: Supplementary file 1 — Additional file 1: Figure S1. Disease incidence of ginger bacterial wilt. Figure S2. The rarefaction curve of samples. Table S1. Soil Physicochemical Data. Table S2. The top ten Phyla of samples. Dataset S1. Discriminative taxa analyzed by LEfSe in all samples. [file 13568_2017_475_MOESM1_ESM.pdf]

Applied Microbiology and Biotechnology Express

Legacy Effects of Continuous Chloropicrin-fumigation for Three-years on Soil Microbial Community Composition and Metabolic Activity

Shuting Zhang[1], Xiaojiao Liu[1,2], Qipeng Jiang[1], Guihua Shen[1], and Wei Ding[1]*

[1] Laboratory of Natural Products Pesticides, College of Plant Protection, Southwest University, Chongqing 400715, China

[2] Department of Microbial Ecology, Netherlands Institute of Ecology, NIOO-KNAW, Wageningen, Netherlands

Corresponding author: Wei Ding
E-mail: dwing818@163.com; fax number: 023-68250218

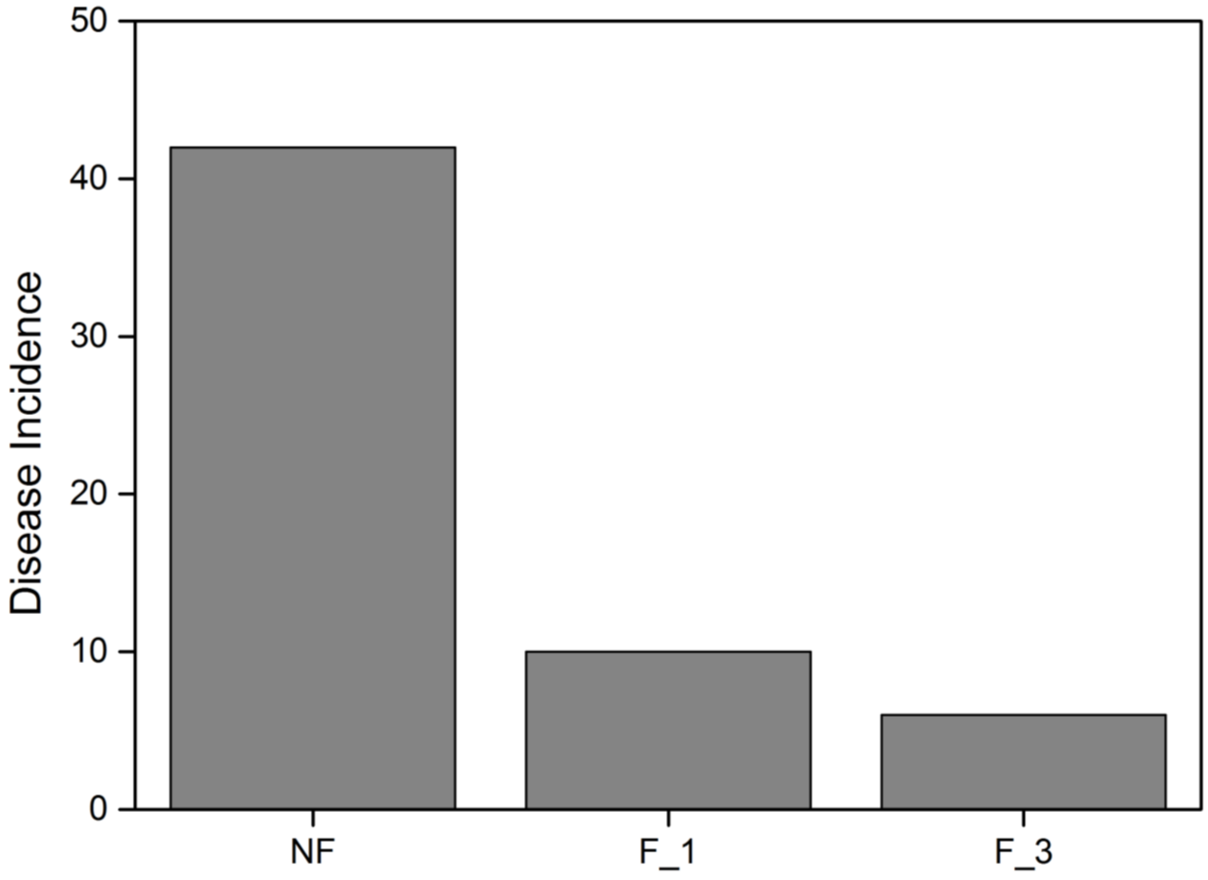

Fig S1 Disease incidence of ginger bacterial wilt

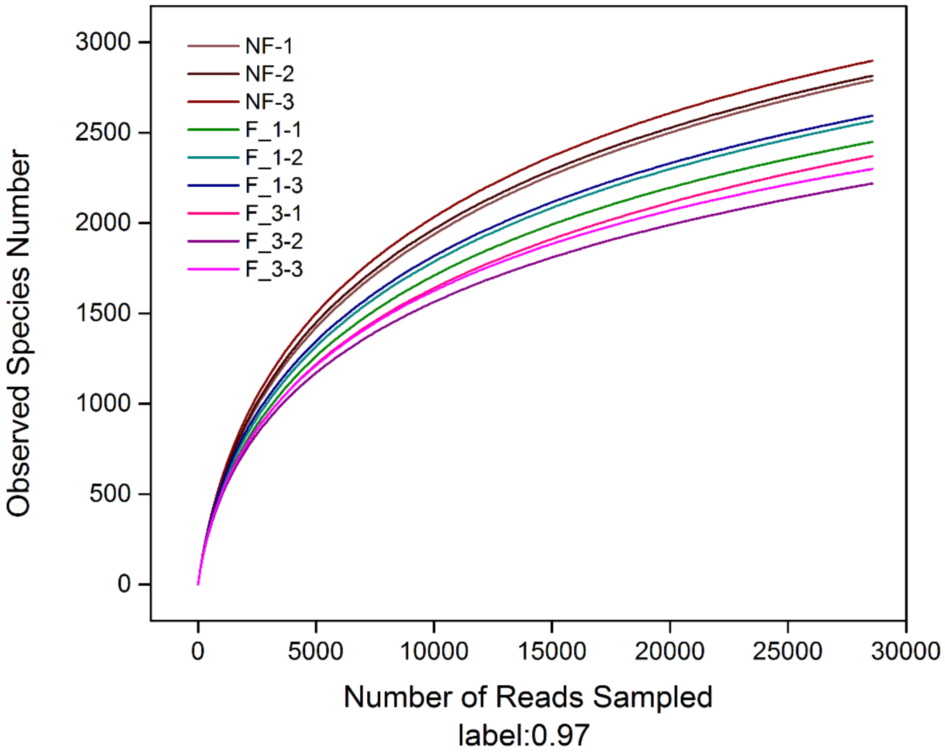

Fig S2 The rarefaction curve of samples

Table S1 Soil Physicochemical Data

	NF	F_1	F_3
pH	5.9	6.6	6.4
Organic matter(g/kg)	13.6	15.8	9.5
Total N(g/kg)	1.04	1.28	1.08
Total P(g/kg)	1.70	2.07	1.38
Total K(g/kg)	17.8	17.4	19.7
Available N(mg/kg)	88.0	84.6	94.8
Available P(mg/kg)	52	144	88
Available K(mg/kg)	290	395	365

Table S2 The top ten Phyla of samples

	NF-Mean(%)	NF-Sd(%)	F_1-Mean(%)	F_1-Sd(%)	F_3-Mean(%)	F_3-Sd(%)	Pvalue	significance sign
Proteobacteria	38.31	3.473	38.8	2.383	38.82	1.828	0.9778	
Bacteroidetes	15.27	2.043	10.27	1.481	11.01	0.2771	0.1063	
Firmicutes	4.819	1.038	12.14	3.797	9.276	1.237	0.02475	*
Acidobacteria	10.93	2.657	6.479	2.444	6.361	1.671	0.1694	
Actinobacteria	6.196	0.3005	6.307	0.8582	8.998	0.4775	0.005473	**
Chloroflexi	8.462	2.605	5.926	1.598	5.237	1.037	0.3026	
Saccharibacteria	3.335	0.3641	4.487	0.1067	8.409	0.802	0.007225	**
Gemmatimonadetes	4.271	0.4868	6.457	0.3533	3.73	0.2394	0.001916	**
Verrucomicrobia	2.365	0.8438	2.605	0.799	2.713	0.403	0.8443	
Planctomycetes	0.779	0.2097	1.328	0.5218	1.155	0.3072	0.2517	

Dataset S1 Discriminative taxa analyzed by LEfSe in all samples

Feature	Log Max Mean Among All the Classes	Top-Rank Class	Log LDA Score	Max Mean
Bacteria.Firmicutes.Bacilli.Bacillales.Paenibacillaceae.Fontibacillus	5.220122067			-
Bacteria.Proteobacteria.Betaproteobacteria.Burkholderiales.Oxalobacteraceae.Pseudoduganella	5.780538329			-
Bacteria.Actinobacteria.Actinobacteria.Streptosporangiales.Streptosporangiaceae.Streptosporangium	5.533696204			-
Bacteria.Firmicutes.Bacilli.Lactobacillales.Streptococcaceae	6.370651396			-
Bacteria.Firmicutes.Clostridia.Clostridiales.Eubacteriaceae	4.30746151			-
Bacteria.Firmicutes.Bacilli.Bacillales.Planococcaceae.Paenisporosarcina	6.211771541			-
Bacteria.Bacteroidetes.Cytophagia.Cytophagales.Flammeovirgaceae.Nafulsella	4.570140022			-
Bacteria.Firmicutes.Bacilli.Bacillales.Bacillaceae.Ureibacillus	4.510199774			-
Bacteria.Actinobacteria.Actinobacteria.Euzebyales	4.702670382	NF	4.474782134	0.022109128
Bacteria.Proteobacteria.Alphaproteobacteria.Rhizobiales.Brucellaceae.Ochrobactrum	6.04812926	F_3	5.746081519	0.027323722
Bacteria.Proteobacteria.Gammaproteobacteria.Oceanospirillales.Oceanospirillaceae.Marinospirillum	4.691909868	NF	4.464346268	0.022109128
Bacteria.Proteobacteria.Gammaproteobacteria.Xanthomonadales.Xanthomonadaceae	6.605806527			-
Bacteria.Proteobacteria.Alphaproteobacteria.Sphingomonadales.Erythrobacteraceae.Altererythrobacter	6.038405727			-
Bacteria.Acidobacteria.Acidobacteria.Holophagales	5.118852073	F_3	4.850568094	0.045938131
Bacteria.Proteobacteria.Gammaproteobacteria.Oceanospirillales.Hahellaceae.Hahella	3.723318839			-
Bacteria.Actinobacteria.Actinobacteria.Micrococcales.Intrasporangiaceae.Ornithinimicrobium	4.207737317	NF	4.429664442	0.022109128
Bacteria.Chloroflexi.Chloroflexia.Kallotenuales	5.39931663			-
Bacteria.Spirochaetae.Spirochaetes.Spirochaetales.Spirochaetaceae	4.310418583			-

Bacteria.Proteobacteria.Deltaproteobacteria.Myxococcales.Cystobacteraceae.Archangium	5.210936043			-
Bacteria.Actinobacteria.Actinobacteria.Kineosporiales	5.476929787	F_3	5.152731945	0.03899022
Bacteria.Bacteroidetes.Sphingobacteriia.Sphingobacteriales.Chitinophagaceae.Lacibacter	4.124938571			-
Bacteria.Actinobacteria.Actinobacteria.Acidimicrobiales.Acidimicrobiaceae	5.603598739			-
Bacteria.Proteobacteria.Betaproteobacteria.Rhodocyclales.Rhodocyclaceae.Azoarcus	4.348464869	NF	4.936962952	0.022109128
Bacteria.Proteobacteria.Gammaproteobacteria.Xanthomonadales.Xanthomonadaceae.Dokdonella	5.770130943	F_3	5.332078446	0.027323722
Bacteria.Chlorobi.Chlorobia.Chlorobiales.OPB56	5.83463262			-
Bacteria.Bacteroidetes.Bacteroidia.Bacteroidales.Bacteroidaceae.Bacteroides	3.948628152			-
Bacteria.Proteobacteria.Alphaproteobacteria.Rhizobiales.Phyllobacteriaceae	6.307586585	F_3	5.820211586	0.027323722
Bacteria.Bacteroidetes.Sphingobacteriia.Sphingobacteriales.Sphingobacteriaceae.Nubsella	4.170476792			-
Bacteria.Actinobacteria.Actinobacteria.Micrococcales.Microbacteriaceae.Agromyces	5.238846409			-
Bacteria.Firmicutes.Bacilli.Bacillales.Bacillaceae.Bacillus	7.324668891			-
Bacteria.Elusimicrobia.Elusimicrobia.Lineage_IIb	5.769030656			-
Bacteria.Elusimicrobia.Elusimicrobia.Lineage_IIa	4.559491783			-
Bacteria.Proteobacteria.Alphaproteobacteria.Rhizobiales.Hyphomicrobiaceae.Rhodoplanes	6.203976345			-
Bacteria.Firmicutes.Bacilli.Bacillales.Planococcaceae.Chungangia	5.022905759			-
Bacteria.Bacteroidetes.Cytophagia.Cytophagales.Cytophagaceae.Rhodocytophaga	5.094867775	NF	4.796608107	0.033985607
Bacteria.Acidobacteria.Acidobacteria.Acidobacteriales.Acidobacteriaceae_Subgroup_1.Telmatobacter	4.218168878			-
Bacteria.Bacteroidetes	5.473660514	F_3	5.152824494	0.027323722
Bacteria.Actinobacteria.Actinobacteria.Corynebacteriales.Nocardiaceae.Rhodococcus	5.654487166	F_3	5.256246096	0.027323722
Bacteria.Firmicutes.Bacilli.Bacillales.Thermoactinomycetaceae.Kroppenstedtia	5.273929572	NF	5.031855642	0.045938131
Bacteria.Acidobacteria.Acidobacteria.Subgroup_3.PAUC26f	5.985440821			-
Bacteria.Firmicutes.Bacilli.Bacillales.Planococcaceae	4.731939173			-

Bacteria.Proteobacteria.Gammaproteobacteria.Order_Incertae_Sedis.Family_Incertae_Sedis.Marinicella	5.810659761			-
Bacteria.Proteobacteria.Gammaproteobacteria.Pseudomonadales.Moraxellaceae	5.776232237	F_3	5.443657752	0.027323722
Bacteria.Acidobacteria.Acidobacteria.Subgroup_10.ABS_19	6.017188005			-
Bacteria.Firmicutes.Clostridia.Clostridiales.Ruminococcaceae	5.140178655			-
Bacteria.Proteobacteria.Gammaproteobacteria.Aeromonadales.Aeromonadaceae.Oceanisphaera	5.528528876			-
Bacteria.Firmicutes.Bacilli.Lactobacillales.Lactobacillaceae.Lactobacillus	5.735115578			-
Bacteria.Bacteroidetes.Flavobacteriia.Flavobacteriales.Flavobacteriaceae	5.156106251			-
Bacteria.Proteobacteria.Gammaproteobacteria.Cellvibrionales.Halieaceae.Haliea	5.494789782	F_1	5.220782867	0.022109128
Bacteria.Firmicutes.Bacilli.Bacillales.Staphylococcaceae.Salinicoccus	5.097828411			-
Bacteria.Proteobacteria.Gammaproteobacteria.Xanthomonadales.Xanthomonadaceae.Luteibacter	3.869446796			-
Bacteria.Proteobacteria.Gammaproteobacteria.Cellvibrionales.Spongiibacteraceae.BD1_7_clade	4.872731695			-
Bacteria.Actinobacteria.Actinobacteria.Micrococcales.Bogoriellaceae.Bogoriella	5.186667503	F_3	4.880360946	0.03899022
Bacteria.Firmicutes.Clostridia.Clostridiales.Family_XI.Sedimentibacter	3.876625464			-
Bacteria.Proteobacteria.Deltaproteobacteria.Bdellovibrionales.Bdellovibrionaceae.OM27_clade	5.829859351			-
Bacteria.Bacteroidetes.Cytophagia.Cytophagales.Cytophagaceae.Dyadobacter	5.319915113	NF	4.828918265	0.03899022
Bacteria.Proteobacteria.Alphaproteobacteria.Sphingomonadales.Sphingomonadaceae.Sphingopyxis	5.798902626			-
Bacteria.Proteobacteria.Alphaproteobacteria.Rhizobiales.DUNssu044	5.100753403			-
Bacteria.Verrucomicrobia.Verrucomicrobiae.Verrucomicrobiales.Verrucomicrobiaceae.Prosthecobacter	5.559545767			-

Bacteria.Actinobacteria.Actinobacteria.Micrococcales.Brevibacteriaceae	6.460233439	F_3	6.138934062	0.027323722
Bacteria.Deinococcus_Thermus.Deinococci	5.827683817			-
Bacteria.Proteobacteria.Gammaproteobacteria.Thiotrichales.EV818SWSAP88	3.647598157			-
Bacteria.Bacteroidetes.Sphingobacteriia.Sphingobacteriales.Sphingobacteriaceae.Sphingobacterium	5.311608339			-
Bacteria.Bacteroidetes.Sphingobacteriia.Sphingobacteriales.KD3_93	6.702391772			-
Bacteria.Proteobacteria.Gammaproteobacteria.Oceanospirillales.Oceanospirillaceae.Oceanobacter	4.112102385			-
Bacteria.Hydrogenedentes	6.315270428			-
Bacteria.Verrucomicrobia.Opitutae	3.885180936			-
Bacteria.Proteobacteria.Deltaproteobacteria.Bdellovibrionales	3.774471411			-
Bacteria.Firmicutes.Clostridia.Clostridiales.Peptococcaceae.Desulfotomaculum	4.505074195			-
Bacteria.Planctomycetes.Phycisphaerae.Phycisphaerales.Phycisphaeraceae.I_8	5.061396098			-
Bacteria.Bacteroidetes.Bacteroidia.Bacteroidales.Porphyromonadaceae.Petrimonas	5.231708605	NF	4.93939154	0.024133897
Bacteria.Firmicutes.Bacilli.Lactobacillales.Carnobacteriaceae	5.646645452			-
Bacteria.Proteobacteria.Gammaproteobacteria.Enterobacteriales	5.448485961			-
Bacteria.Proteobacteria.Alphaproteobacteria.Rhodospirillales.Acetobacteraceae.Acidocella	4.425447148	F_1	4.503095494	0.022109128
Bacteria.Proteobacteria.Gammaproteobacteria.Legionellales.Coxiellaceae.Coxiella	5.576779836			-
Bacteria.Actinobacteria.Actinobacteria.Acidimicrobiales.Acidimicrobiales_Incertae_Sedis.Aciditerrimonas	5.55970393	F_3	5.27031975	0.027323722
Bacteria.Proteobacteria.Deltaproteobacteria.Myxococcales.Phaselicystidaceae.Phaselicystis	5.388892428			-
Bacteria.Proteobacteria.Gammaproteobacteria.Pseudomonadales.Moraxellaceae.Acinetobacter	5.04212318			-
Bacteria.Proteobacteria.Alphaproteobacteria.Rhodospirillales.CCU22	5.03821273			-
Bacteria.Planctomycetes.Phycisphaerae.WD2101_soil_group	6.058343658			-

Bacteria.Firmicutes.Clostridia.Clostridiales.Family_XVIII	5.695972361			-
Bacteria.Actinobacteria.Actinobacteria.Gaiellales.Gaiellaceae	6.0515098	NF	5.696493103	0.027323722
Bacteria.Proteobacteria.Gammaproteobacteria.Xanthomonadales.Xanthomonadaceae.Pseudoxanthomonas	5.99596			-
Bacteria.Actinobacteria.Actinobacteria.Catenulisporales.Catenulisporaceae.Catenulispora	4.170476792			-
Bacteria.Firmicutes.Erysipelotrichia	5.614558326	F_1	5.221160722	0.03899022
Bacteria.Proteobacteria.Alphaproteobacteria.Rhizobiales	6.282756373	F_3	5.848582435	0.03899022
Bacteria.Verrucomicrobia.OPB35_soil_group	6.797943194			-
Bacteria.Chlamydiae.Chlamydiae.Chlamydiales.Waddliaceae	5.548152102	F_3	5.259519965	0.024133897
Bacteria.Planctomycetes.Phycisphaerae	6.418796249			-
Bacteria.Actinobacteria.Actinobacteria.Micrococcales.Demequinaceae.Demequina	4.792325438			-
Bacteria.Actinobacteria.Actinobacteria.Streptosporangiales.Thermomonosporaceae.Actinocorallia	4.920790352			-
Bacteria.Proteobacteria.Alphaproteobacteria.Rhodospirillales.Acetobacteraceae	5.335526869			-
Bacteria.Firmicutes.Bacilli.Bacillales.Bacillaceae.Amphibacillus	5.794314594	F_3	5.489270217	0.0265095
Bacteria.Firmicutes.Bacilli.Lactobacillales	6.762096724			-
Bacteria.Proteobacteria.Alphaproteobacteria.Rhizobiales.FFCH5858	4.518761977			-
Bacteria.Actinobacteria.Actinobacteria.Micrococcales.Bogoriellaceae	5.908655232	F_3	5.596957596	0.027323722
Bacteria.Proteobacteria.Betaproteobacteria.Burkholderiales.Oxalobacteraceae	5.907519379			-
Bacteria.Actinobacteria.Actinobacteria.Catenulisporales.Catenulisporaceae	4.170476792			-
Bacteria.Verrucomicrobia.Spartobacteria	6.202532129			-
Bacteria.Acidobacteria.Acidobacteria.Acidobacteriales.Acidobacteriaceae_Subgroup_1.Granulicella	4.629102013			-
Bacteria.Proteobacteria.Gammaproteobacteria.Thiotrichales	5.2998793	F_3	4.969149823	0.03899022
Bacteria.Verrucomicrobia.Spartobacteria.Chthoniobacterales	4.634916788			-

Bacteria.Proteobacteria.Gammaproteobacteria.Xanthomonadales.Nevskiaceae.Alkanibacter	4.820597153			-
Bacteria.Proteobacteria.Deltaproteobacteria.Myxococcales.Sandaracinaceae	5.813884467			-
Bacteria.Bacteroidetes.Sphingobacteriia.Sphingobacteriales.Chitinophagaceae.Segetibacter	4.982912979	NF	4.691898511	0.034735259
Bacteria.Proteobacteria.Gammaproteobacteria.Xanthomonadales.Xanthomonadaceae.Ignatzschineria	4.974324159	NF	4.70255972	0.022109128
Bacteria.Proteobacteria.Betaproteobacteria.Burkholderiales.Burkholderiaceae	5.772121135			-
Bacteria.Actinobacteria.Actinobacteria.Gaiellales	5.679730738			-
Bacteria.Candidate_division_SR1	4.050677799			-
Bacteria.Proteobacteria.Gammaproteobacteria.Chromatiales.Chromatiaceae.Rheinheimera	4.72886478			-
Bacteria.Proteobacteria.Gammaproteobacteria.Cellvibrionales.Cellvibrionaceae.Cellvibrio	5.891814655			-
Bacteria.Firmicutes.Bacilli.Bacillales.Bacillaceae.Paucisalibacillus	6.231630008			-
Bacteria.Proteobacteria.Gammaproteobacteria.Cellvibrionales.Cellvibrionaceae	3.723318839			-
Bacteria.Planctomycetes.Phycisphaerae.Phycisphaerales.Phycisphaeraceae.AKYG587	4.836784895	NF	4.545233945	0.034863256
Bacteria.Bacteroidetes.Sphingobacteriia.Sphingobacteriales.AKYH767	4.473014935			-
Bacteria.Acidobacteria.Acidobacteria	4.754036206	NF	4.547337616	0.045938131
Bacteria.Proteobacteria.Alphaproteobacteria.Rhodospirillales.ML80	3.61956934			-
Bacteria.Firmicutes.Erysipelotrichia.Erysipelotrichales.Erysipelotrichaceae.Turicibacter	5.607777626			-
Bacteria.Proteobacteria.Alphaproteobacteria.Rhodospirillales.AT_s3_44	4.381366849			-
Bacteria.Proteobacteria.Gammaproteobacteria.Cellvibrionales.Porticoccaceae	4.826403365			-
Bacteria.Actinobacteria.Actinobacteria.Corynebacteriales.Segniliparaceae.Segniliparus	3.948628152			-
Bacteria.Proteobacteria.Alphaproteobacteria.Rickettsiales.Rickettsiaceae.Candidatus_Trichorickettsia	4.841637349			-
Bacteria.Bacteroidetes.Cytophagia.Cytophagales.Flammeovirgaceae.Reichenbachiella	4.383703331			-
Bacteria.Proteobacteria.Alphaproteobacteria.Rhodobacterales.Rhodobacteraceae.Rubellimicrobium	5.444757841			-

Bacteria.Bacteroidetes.Bacteroidia.Bacteroidales.Porphyromonadaceae.Proteiniphilum	5.378195797	NF	5.061564582	0.034735259
Bacteria.Proteobacteria.Alphaproteobacteria.Rhizobiales.Rhizobiales_Incertae_Sedis.Rhizomicrobium	6.967103284	F_3	6.644683508	0.027323722
Bacteria.Actinobacteria.Actinobacteria.Rubrobacterales.Rubrobacteriaceae.Rubrobacter	4.709563093	NF	4.591032432	0.037941542
Bacteria.Proteobacteria.Alphaproteobacteria.Rhizobiales.Methylobacteriaceae.Methylobacterium	4.505074195			-
Bacteria.Proteobacteria.Alphaproteobacteria.Rhizobiales.Hyphomicrobiaceae.Devosia	6.788791044			-
Bacteria.Firmicutes.Erysipelotrichia.Erysipelotrichales.Erysipelotrichaceae	5.614558326	F_1	5.167888744	0.03899022
Bacteria.Proteobacteria.Gammaproteobacteria.Xanthomonadales.Xanthomonadaceae.Xanthomonas	4.427439767			-
Bacteria.Proteobacteria.Betaproteobacteria.Burkholderiales.Comamonadaceae.Hylemonella	4.719143107	F_3	4.736513276	0.022109128
Bacteria.Proteobacteria.Gammaproteobacteria.Xanthomonadales.Xanthomonadales_Incertae_Sedis.Steroidobacter	6.840743181			-
Bacteria.Proteobacteria.Alphaproteobacteria.Rickettsiales.Mitochondria	4.906095636			-
Bacteria.Proteobacteria.Deltaproteobacteria.Myxococcales.Elev_16S_1158	4.450382463			-
Bacteria.Proteobacteria.Alphaproteobacteria.Rhizobiales.Bradyrhizobiaceae.Afipia	4.986134848			-
Bacteria.Proteobacteria.Alphaproteobacteria.Sphingomonadales.Sphingomonadaceae.Sphingomonas	7.409700587			-
Bacteria.Actinobacteria.Actinobacteria.Streptomycetales.Streptomycetaceae.Streptomyces	6.166213909			-
Bacteria.Acidobacteria.Acidobacteria.Subgroup_3.SJA_149	5.923156261			-
Bacteria.Proteobacteria.Gammaproteobacteria.Pseudomonadales.Pseudomonadaceae	6.572333003			-
Bacteria.Proteobacteria.Gammaproteobacteria.Pseudomonadales.Pseudomonadaceae.Pseudomonas	6.572333003			-
Bacteria.Proteobacteria.Alphaproteobacteria.Sphingomonadales.7B_8	5.539447626			-
Bacteria.Proteobacteria.Deltaproteobacteria.Myxococcales.Nannocystaceae.Nannocystis	6.199826266			-

Bacteria.Proteobacteria.Gammaproteobacteria.Xanthomonadales.Xanthomonadaceae.Panacagrimonas	5.522758046			-
Bacteria.Proteobacteria.Deltaproteobacteria.Myxococcales.mle1_27	5.252133957			-
Bacteria.Actinobacteria.Actinobacteria.Rubrobacterales.Rubrobacteriaceae	4.709563093	NF	4.58356382	0.037941542
Bacteria.Bacteroidetes.Sphingobacteriia.Sphingobacteriales.Sphingobacteriaceae.Parapedobacter	6.013182908			-
Bacteria.Chloroflexi.Thermomicrobia.Sphaerobacterales	6.523429532	F_3	6.213979017	0.03899022
Bacteria.Bacteroidetes.Bacteroidetes_VC2_1_Bac22	5.638277973	F_3	5.097630282	0.027323722
Bacteria.Firmicutes.Bacilli.Bacillales.Thermoactinomycetaceae.Planifilum	3.98282689			-
Bacteria.Proteobacteria.Betaproteobacteria.Neisseriales.Neisseriaceae	4.505074195			-
Bacteria.Proteobacteria.Gammaproteobacteria.Xanthomonadales.Xanthomonadaceae.Luteimonas	6.733127169	F_3	6.452421174	0.027323722
Bacteria.Proteobacteria.Alphaproteobacteria.Rhizobiales.Rhodobiaceae.Parvibaculum	5.074177455			-
Bacteria.Proteobacteria.Alphaproteobacteria.Rhizobiales.A0839	5.247912638	NF	4.927341233	0.049647804
Bacteria.Proteobacteria.Deltaproteobacteria.Desulfobacterales.Desulfobulbaceae	3.4892356			-
Bacteria.Bacteroidetes.Sphingobacteriia.Sphingobacteriales.Chitinophagaceae.Crenotalea	4.711402817			-
Bacteria.Firmicutes.Clostridia.Clostridiales.Clostridiaceae_2	4.505074195			-
Bacteria.Firmicutes.Clostridia.Clostridiales.Clostridiaceae_3	3.61956934			-
Bacteria.Proteobacteria.Gammaproteobacteria.Xanthomonadales.Xanthomonadaceae.Arenimonas	6.376571319			-
Bacteria.Firmicutes.Clostridia.Clostridiales.Clostridiaceae_1	3.497353564			-
Bacteria.Proteobacteria.Gammaproteobacteria.Alteromonadales.Idiomarinaceae	4.597004374			-
Bacteria.Planctomycetes.Phycisphaerae.Phycisphaerales	6.148495571			-
Bacteria.Firmicutes.Bacilli.Bacillales.Bacillaceae.Oceanobacillus	5.902802699	F_3	5.562391571	0.03899022
Bacteria.Proteobacteria.Alphaproteobacteria.Rhodospirillales.Rhodospirillaceae.Skermanella	5.517008481	NF	5.193210597	0.045938131

Bacteria.Bacteroidetes.Sphingobacteriia.Sphingobacteriales.Sphingobacteriaceae.Mucilaginibacter	5.516979359			-
Bacteria.Bacteroidetes.Sphingobacteriia.Sphingobacteriales.Chitinophagaceae	6.963819055	F_3	6.608614704	0.027323722
Bacteria.Verrucomicrobia.UA11	4.678902161			-
Bacteria.Proteobacteria.Alphaproteobacteria.DB1_14	5.906594414			-
Bacteria.Acidobacteria.Acidobacteria.Subgroup_4.11_24	5.469906545			-
Bacteria.Firmicutes.Bacilli.Lactobacillales.Streptococcaceae.Streptococcus	5.32062062			-
Bacteria.Firmicutes.Clostridia.Clostridiales.Clostridiaceae_1.Clostridium_sensu_stricto_5	3.869446796			-
Bacteria.Chlamydiae.Chlamydiae.Chlamydiales	5.114452733			-
Bacteria.Proteobacteria.Deltaproteobacteria.Syntrophobacterales	4.313503427	NF	4.504438948	0.022109128
Bacteria.Proteobacteria.Deltaproteobacteria.Desulfuromonadales.GR_WP33_58	4.793945871			-
Bacteria.Firmicutes.Clostridia.Clostridiales.Peptococcaceae.Thermincola	4.337183656			-
Bacteria.Actinobacteria.Actinobacteria.Kineosporiales.Kineosporiaceae.Angustibacter	5.476929787	F_3	5.164229811	0.027323722
Bacteria.Proteobacteria.Alphaproteobacteria.Caulobacterales.Hyphomonadaceae.Hirschia	5.620615184			-
Bacteria.Actinobacteria.Actinobacteria.Streptomycetales	6.166213909			-
Bacteria.Proteobacteria.Alphaproteobacteria.Rhizobiales.MNG7	5.424659897			-
Bacteria.Cyanobacteria.Cyanobacteria.Vampirovibrionales	5.606631508	F_3	5.271332648	0.03899022
Bacteria.Proteobacteria.Betaproteobacteria.Burkholderiales.Comamonadaceae.Aquabacterium	4.654167479	F_3	4.572494054	0.045938131
Bacteria.Verrucomicrobia.Verrucomicrobiae.Verrucomicrobiales.Verrucomicrobiaceae.Roseimicrobium	5.198894628			-
Bacteria.Verrucomicrobia.Spartobacteria.Chthoniobacterales.Chthoniobacteraceae	5.984318909			-
Bacteria.Cyanobacteria.Cyanobacteria.SubsectionIV	4.971574292			-
Bacteria.Actinobacteria.Actinobacteria.Actinomycetales.Actinomycetaceae.Flaviflexus	5.150049761	F_3	4.890578769	0.033985607
Bacteria.Candidate_division_OP3	5.237565297			-
Bacteria.Firmicutes.Bacilli.Bacillales.Bacillaceae.Sinibacillus	4.997445763	NF	4.696977684	0.022109128

Bacteria.Bacteroidetes.Flavobacteriia.Flavobacteriales.Cryomorphaceae.Fluviicola	4.921814024			-
Bacteria.Bacteroidetes.Flavobacteriia.Flavobacteriales.NS9_marine_group	5.009019992			-
Bacteria.Bacteroidetes.Cytophagia.Cytophagales.Cytophagaceae.Sporocytophaga	5.940099921			-
Bacteria.Proteobacteria.Gammaproteobacteria.Pseudomonadales	6.601705452			-
Bacteria.Firmicutes.Clostridia.Clostridiales.Family_XI.Anaerosalibacter	4.183613712			-
Bacteria.Proteobacteria.Betaproteobacteria.Neisseriales.Neisseriaceae.Paludibacterium	4.837940922	NF	4.553222542	0.022109128
Bacteria.Planctomycetes.Planctomycetacia.Planctomycetales.Planctomycetaceae.Planctomyces	5.221769108			-
Bacteria.Chloroflexi.Chloroflexia.Chloroflexales.Chloroflexaceae.Candidatus_Chloroploca	3.723318839			-
Bacteria.Firmicutes.Bacilli.Lactobacillales.Carnobacteriaceae.Trichococcus	5.27192771			-
Bacteria.Actinobacteria.Actinobacteria.Actinomycetales	5.150049761	F_3	4.898607269	0.033985607
Bacteria.Proteobacteria.Alphaproteobacteria.Rhodospirillales.DA111	5.491991277	F_3	5.047981202	0.027323722
Bacteria.Actinobacteria.Actinobacteria.Pseudonocardiales.Pseudonocardiaceae.Saccharothrix	4.38590039			-
Bacteria.Proteobacteria.Alphaproteobacteria.Rhizobiales.Xanthobacteraceae	6.34694257			-
Bacteria.Chlamydiae.Chlamydiae.Chlamydiales.Simkaniaceae.Candidatus_Rhabdochlamydia	6.147480027	F_3	5.825042147	0.027323722
Bacteria.Bacteroidetes.Flavobacteriia.Flavobacteriales.Flavobacteriaceae.Galbibacter	5.26386774			-
Bacteria.Planctomycetes.Phycisphaerae.CPla_3_termite_group	4.854620218			-
Bacteria.Proteobacteria.Gammaproteobacteria.Xanthomonadales.Xanthomonadales_Incertae_Sedis.Acidibacter	6.162005382			-
Bacteria.Chloroflexi.Anaerolineae.Anaerolineales.Anaerolineaceae.Anaerolinea	4.28372367	NF	4.456841523	0.022109128
Bacteria.Firmicutes.Clostridia.Clostridiales.Defluviitaleaceae	4.181000866			-
Bacteria.Proteobacteria.Alphaproteobacteria.Rickettsiales.Rickettsiaceae	5.112694909	F_3	4.923227184	0.0265095
Bacteria.Proteobacteria.Alphaproteobacteria.Rhodospirillales.AKYH478	5.668186954			-
Bacteria.Firmicutes.Bacilli.Bacillales.Planococcaceae.Bhargavaea	4.326952098			-
Bacteria.Proteobacteria.Alphaproteobacteria.Rhizobiales.Hyphomicrobiaceae.Pelagibacterium	4.170476792			-
Bacteria.Firmicutes.Clostridia.Clostridiales.Lachnospiraceae.Mobilitalea	5.039927753			-

Bacteria.Bacteroidetes.Flavobacteriia.Flavobacteriales.Flavobacteriaceae.Salinimicrobium	5.985751912			-
Bacteria.Proteobacteria.Gammaproteobacteria.Oceanospirillales.Oceanospirillaceae.Pseudospirillum	6.086974676	NF	5.68937768	0.03899022
Bacteria.Acidobacteria.Acidobacteria.Acidobacteriales.Acidobacteriaceae_Subgroup_1.Acidobacterium	5.718075398	F_3	5.435542517	0.024133897
Bacteria.Actinobacteria.Actinobacteria.Bifidobacteriales.Bifidobacteriaceae.Bifidobacterium	4.69780032			-
Bacteria.Bacteroidetes.Cytophagia.Cytophagales.Cytophagaceae.Ohtaekwangia	6.124355258			-
Bacteria.Proteobacteria.Alphaproteobacteria.Rhodospirillales.Acetobacteraceae.Rubritepida	4.524455826	NF	4.432323067	0.022109128
Bacteria.Proteobacteria.Betaproteobacteria.Burkholderiales.Burkholderiaceae.Limnobacter	5.224014834			-
Bacteria.Cyanobacteria.Cyanobacteria.Obscuribacterales	5.63482914			-
Bacteria.Firmicutes.Clostridia.Clostridiales.Peptostreptococcaceae.Terrisporobacter	4.664225571			-
Bacteria.Proteobacteria.Alphaproteobacteria.Rhizobiales.Rhizobiales_Incertae_Sedis.Alsobacter	4.595598649			-
Bacteria.Proteobacteria.Alphaproteobacteria.Sphingomonadales.Erythrobacteraceae.Erythrobacter	4.390771792			-
Bacteria.Actinobacteria.Actinobacteria.Streptosporangiales	4.84947993			-
Bacteria.Acidobacteria.Acidobacteria.Holophagales.Holophagaceae	5.118852073	F_3	4.869654972	0.045938131
Bacteria.Proteobacteria.Deltaproteobacteria.Myxococcales.bacteriap25	4.374154447	NF	4.381606056	0.022109128
Bacteria.Bacteroidetes.Cytophagia.Cytophagales.Cytophagaceae.Persicitalea	4.595598649			-
Bacteria.Proteobacteria.Alphaproteobacteria.Caulobacterales.Caulobacteraceae.Caulobacter	5.819752801			-
Bacteria.Proteobacteria.Alphaproteobacteria.Rhizobiales.D05_2	3.4892356			-
Bacteria.Actinobacteria.Actinobacteria.Frankiales.Sporichthyaceae	4.839963299			-
Bacteria.Proteobacteria.Alphaproteobacteria.Rhizobiales.Xanthobacteraceae.Pseudolabrys	6.159899664	F_3	5.698380145	0.027323722
Bacteria.Firmicutes.Bacilli.Bacillales.Planococcaceae.Solibacillus	5.253450755			-
Bacteria.Bacteroidetes.Sphingobacteriia.Sphingobacteriales.Chitinophagaceae.Ferruginibacter	4.453194357			-

Bacteria.Proteobacteria.Alphaproteobacteria.Rhodospirillales.Acetobacteraceae.Rhodovastum	5.788650743	F_3	5.478364168	0.027323722
Bacteria.Proteobacteria.Betaproteobacteria.Hydrogenophilales.Hydrogenophilaceae.Thiobacillus	4.3352524			-
Bacteria.Firmicutes.Bacilli.Bacillales.Sporolactobacillaceae.Tuberibacillus	4.802721304			-
Bacteria.Bacteroidetes.Flavobacteriia.Flavobacteriales.Flavobacteriaceae.Elizabethkingia	4.887308824			-
Bacteria.Proteobacteria.Gammaproteobacteria.Aeromonadales	5.542745791			-
Bacteria.Cyanobacteria.Cyanobacteria.SubsectionIII.FamilyI.Leptolyngbya	5.339856245	NF	5.029463702	0.045938131
Bacteria.Proteobacteria.Gammaproteobacteria.Oceanospirillales.Halomonadaceae	5.1257995	F_3	4.877057608	0.027323722
Bacteria.Proteobacteria.Deltaproteobacteria.Sh765B_TzT_29	5.553088399			-
Bacteria.Actinobacteria.Actinobacteria.Micrococcales.Micrococcaceae	5.433443763			-
Bacteria.Proteobacteria.Betaproteobacteria.Burkholderiales.Comamonadaceae.Comamonas	4.30846588	F_1	4.497358076	0.022109128
Bacteria.Proteobacteria.Alphaproteobacteria.Rhodospirillales.Acetobacteraceae.Roseomonas	5.836139596			-
Bacteria.Proteobacteria.Deltaproteobacteria.Desulfurellales	3.647598157			-
Bacteria.Proteobacteria.Alphaproteobacteria.Rhodospirillales.Acetobacteraceae.Craurococcus	4.847960708			-
Bacteria.Proteobacteria.Alphaproteobacteria.OCS116_clade	4.85620254	F_1	4.622530633	0.034863256
Bacteria.Chloroflexi.Thermomicrobia.AKYG1722	6.166527253	F_3	5.720932821	0.027323722
Bacteria.Bacteroidetes.Sphingobacteriia.Sphingobacteriales.Chitinophagaceae.Flavitalea	5.936258405			-
Bacteria.Actinobacteria.Actinobacteria.Frankiales.Frankiaceae.Jatrophihabitans	4.390771792	F_3	5.080482248	0.034735259
Bacteria.Proteobacteria.Gammaproteobacteria.Pseudomonadales.Moraxellaceae.Psychrobacter	5.378083722			-
Bacteria.Planctomycetes.Planctomycetacia.Planctomycetales.Planctomycetaceae.Singulisphaera	6.299072388	F_3	5.974836693	0.027323722
Bacteria.Firmicutes.Bacilli.Bacillales.Thermoactinomycetaceae.Shimazuella	5.010098287			-
Bacteria.Nitrospirae.Nitrospira	6.456736143	NF	6.073087664	0.03899022
Bacteria.Firmicutes.Clostridia.Clostridiales.Defluviitaleaceae.Defluviitalea	4.181000866			-

Bacteria.Chloroflexi.Chloroflexia.Herpetosiphonales.Herpetosiphonaceae.Herpetosiphon	5.240403989			-
Bacteria.Proteobacteria.Betaproteobacteria.Burkholderiales.Burkholderiaceae.Cupriavidus	5.214222611			-
Bacteria.Verrucomicrobia	7.165671301			-
Bacteria.Actinobacteria.Actinobacteria.Corynebacteriales.Dietziaceae.Dietzia	5.72168855	F_3	5.401749253	0.027323722
Bacteria.Proteobacteria.Alphaproteobacteria.Rhizobiales.Beijerinckiaceae.Chelatococcus	5.11096257			-
Bacteria.Proteobacteria.Alphaproteobacteria.Rhodospirillales.Acetobacteraceae.Stella	4.843242365			-
Bacteria.Acidobacteria.Acidobacteria.Acidobacteriales.Acidobacteriaceae_Subgroup_1.Terriglobus	5.065466845	F_3	4.837423883	0.022109128
Bacteria.Acidobacteria.Acidobacteria.Subgroup_10.NS72	4.136766684			-
Bacteria.Proteobacteria.Gammaproteobacteria.Oceanospirillales	6.110543655			-
Bacteria.Proteobacteria.Deltaproteobacteria.Myxococcales.Phaselicystidaceae	5.388892428			-
Bacteria.Actinobacteria.Actinobacteria.Frankiales.Nakamurellaceae	4.650284467			-
Bacteria.Proteobacteria.Betaproteobacteria.Burkholderiales.Comamonadaceae.Methylibium	5.463703674	F_3	5.081848005	0.03899022
Bacteria.Actinobacteria.Actinobacteria.Kineosporiales.Kineosporiaceae.Quadrisphaera	3.61956934			-
Bacteria.Proteobacteria.Gammaproteobacteria.Order_Incertae_Sedis.Family_Incertae_Sedis	5.810659761			-
Bacteria.Firmicutes.Bacilli.Lactobacillales.Aerococcaceae	6.340853094	F_3	5.979863874	0.03899022
Bacteria.Proteobacteria.Gammaproteobacteria.Cellvibrionales.Halieaceae.OM60_NOR5_clade	5.886102467	F_3	5.620032461	0.0265095
Bacteria.Planctomycetes.OM190	6.023836619	F_1	5.614861036	0.027323722
Bacteria.Firmicutes.Clostridia.Clostridiales	5.266052613	F_3	4.995986642	0.024133897
Bacteria.Proteobacteria.Betaproteobacteria.TRA3_20	6.677623846			-
Bacteria.Proteobacteria.Alphaproteobacteria.Rickettsiales.TK34	5.204287681			-
Bacteria.Planctomycetes.Planctomycetacia.Planctomycetales.Planctomycetaceae.Isosphaera	5.427903232	F_3	5.143529267	0.0265095
Bacteria.Actinobacteria.Actinobacteria.Micrococcales.Bogoriellaceae.Georgenia	4.738396043			-
Bacteria.Bacteroidetes.Sphingobacteriia.Sphingobacteriales.env_OPS_17	6.460591635	F_3	5.943299804	0.03899022

Bacteria.Proteobacteria.Gammaproteobacteria.Legionellales.Coxiellaceae	5.229580692	NF	4.956944406	0.034735259
Bacteria.Actinobacteria.Actinobacteria.Micromonosporales.Micromonosporaceae.Micromonospora	6.2946936			-
Bacteria.Bacteroidetes.Flavobacteriia.Flavobacteriales.Flavobacteriaceae.Gelidibacter	4.669481364			-
Bacteria.Proteobacteria.Gammaproteobacteria.Xanthomonadales.Xanthomonadaceae.Stenotrophomonas	4.892663203			-
Bacteria.Actinobacteria.Actinobacteria.Acidimicrobiales.Acidimicrobiaceae.Illumatobacter	5.911782131	NF	5.550497045	0.027323722
Bacteria.Firmicutes.Bacilli.Bacillales.Bacillaceae.Fictibacillus	5.601237668	F_1	5.256414911	0.027323722
Bacteria.Actinobacteria.Actinobacteria.Frankiales	5.983967286			-
Bacteria.Chloroflexi.Chloroflexia.Chloroflexales.Roseiflexaceae.Roseiflexus	6.305999296			-
Bacteria.Proteobacteria.Alphaproteobacteria.Rhizobiales.Rhizobiaceae	6.385658313	F_3	5.93783761	0.03899022
Bacteria.Proteobacteria.Alphaproteobacteria.Rhodobacterales	6.01457444			-
Bacteria.Proteobacteria.Gammaproteobacteria.Cellvibrionales	6.35038861	F_3	5.941526591	0.027323722
Bacteria.Firmicutes.Bacilli.Bacillales.Paenibacillaceae.Paenibacillus	6.631213269			-
Bacteria.Verrucomicrobia.Opitutae.Opitutales.Opitutaceae	6.683140002	F_3	6.258036942	0.027323722
Bacteria.Proteobacteria.Alphaproteobacteria.Rhizobiales.Bradyrhizobiaceae	5.966141686	F_3	5.596887127	0.03899022
Bacteria.Actinobacteria.Actinobacteria.Solirubrobacterales.Patulibacteraceae.Patulibacter	5.369206282			-
Bacteria.Actinobacteria.Actinobacteria.Propionibacteriales.Nocardioidaceae.Aeromicrobium	6.26321947	NF	5.744761877	0.03899022
Bacteria.Proteobacteria.Alphaproteobacteria.Caulobacterales.Caulobacteraceae	6.265273418			-
Bacteria.Proteobacteria.Betaproteobacteria.Nitrosomonadales	4.680066593			-
Bacteria.Proteobacteria.Alphaproteobacteria.Rhizobiales.Methylobacteriaceae	6.520164568	F_3	6.173158635	0.027323722
Bacteria.Bacteroidetes.Cytophagia.Cytophagales.Cytophagaceae.Cytophaga	4.399078001			-
Bacteria.Actinobacteria.Actinobacteria.Frankiales.Sporichthyaceae.Sporichthya	4.839963299			-
Bacteria.Proteobacteria.Betaproteobacteria.Burkholderiales.Oxalobacteraceae.Undibacterium	4.228969626			-
Bacteria.Bacteroidetes.Cytophagia.Cytophagales.Cytophagaceae.Hymenobacter	4.343025912	NF	4.458803592	0.022109128

Bacteria.Spirochaetae.Spirochaetes.Spirochaetales.Leptospiraceae	4.497554413			-
Bacteria.Bacteroidetes.Sphingobacteriia.Sphingobacteriales.Chitinophagaceae.Terrimonas	6.248356048			-
Bacteria.Proteobacteria.Alphaproteobacteria.Rhizobiales.Brucellaceae	6.04812926	F_3	5.743533314	0.027323722
Bacteria.WCHB1_60	5.693059028	NF	5.347936724	0.027323722
Bacteria.Chloroflexi.Ktedonobacteria	5.434078272			-
Bacteria.Proteobacteria.Alphaproteobacteria.Rhodospirillales.Rhodospirillaceae.Pelagibius	4.014280396			-
Bacteria.Proteobacteria.Alphaproteobacteria.Rhodospirillales.Rhodospirillaceae.Elstera	3.774471411			-
Bacteria.Proteobacteria.Deltaproteobacteria.Bdellovibrionales.Bacteriovoracaceae.Peredibacter	5.132512704			-
Bacteria.Proteobacteria.Deltaproteobacteria.43F_1404R	3.774471411			-
Bacteria.Proteobacteria.Epsilonproteobacteria.BG_g7	4.435822379	NF	4.323887005	0.022109128
Bacteria.Acidobacteria.Acidobacteria.Acidobacteriales.Acidobacteriaceae_Subgroup_1.Edaphobacter	4.519339811			-
Bacteria.Proteobacteria.Gammaproteobacteria.Cellvibrionales.Spongiibacteraceae	4.872731695			-
Bacteria.Proteobacteria.Alphaproteobacteria.Rickettsiales.Anaplasmataceae	5.78402707	F_1	5.526003399	0.034735259
Bacteria.Firmicutes.Clostridia.Clostridiales.Family_XI.Tissierella	5.578147824			-
Bacteria.Actinobacteria.Actinobacteria.Corynebacteriales.Nocardiaceae.Nocardia	4.351707795			-
Bacteria.Firmicutes.Clostridia.Clostridiales.Family_XI	4.770785374			-
Bacteria.Firmicutes.Clostridia.Clostridiales.Peptostreptococcaceae.Romboutsia	5.323227059			-
Bacteria.Chlamydiae.Chlamydiae.Chlamydiales.Parachlamydiaceae.Neochlamydia	6.144771825			-
Bacteria.Firmicutes.Bacilli.Bacillales.Alicyclobacillaceae.Tumebacillus	6.046877928			-
Bacteria.Verrucomicrobia.Opitutae.Opitutales.Opitutaceae.Alterococcus	4.427439767	NF	4.347475608	0.022109128
Bacteria.Proteobacteria.Gammaproteobacteria.34P16	5.263281607			-
Bacteria.Proteobacteria.Gammaproteobacteria.Xanthomonadales.Xanthomonadaceae.Chiayiivirga	5.330188105			-

Bacteria.Actinobacteria.Actinobacteria.Solirubrobacterales.Conexibacteraceae.Conexibacter	5.356501503			-
Bacteria.Bacteroidetes.Sphingobacteriia.Sphingobacteriales.Sphingobacteriaceae	6.342657607	F_3	6.020167398	0.027323722
Bacteria.Bacteroidetes.Sphingobacteriia.Sphingobacteriales.Chitinophagaceae.Chitinophaga	6.325000605	F_3	6.018249541	0.027323722
Bacteria.Proteobacteria.Alphaproteobacteria.Rhizobiales.KF_JG30_B3	5.667164636	NF	5.206117769	0.03899022
Bacteria.Proteobacteria.Gammaproteobacteria.Legionellales.Coxiellaceae.Aquicella	7.192298939			-
Bacteria.Bacteroidetes.Flavobacteriia.Flavobacteriales.Cryomorphaceae.Crocinitomix	5.540954906			-
Bacteria.Firmicutes.Clostridia.Clostridiales.Family_XI.Gallicola	4.632134679			-
Bacteria.Proteobacteria.Alphaproteobacteria.Rickettsiales.LWSR_14	5.101018027			-
Bacteria.Firmicutes.Bacilli.Bacillales.Planococcaceae.Rummeliibacillus	4.840810075			-
Bacteria.Proteobacteria.Betaproteobacteria.Methylophilales	6.346055529	NF	5.823757481	0.03899022
Bacteria.Proteobacteria.Gammaproteobacteria.Xanthomonadales	6.368843376			-
Bacteria.Proteobacteria.Betaproteobacteria.Burkholderiales.Comamonadaceae	6.217540822	NF	5.779575432	0.027323722
Bacteria.Proteobacteria.Alphaproteobacteria.Rhizobiales.Methylobacteriaceae.Meganema	4.63558208	F_1	4.613775691	0.034735259
Bacteria.Actinobacteria.Actinobacteria.Frankiales.Geodermatophilaceae	5.539367769			-
Bacteria.Proteobacteria.TA18	5.489889119			-
Bacteria.Proteobacteria.Alphaproteobacteria.Rhizobiales.Rhizobiaceae.Shinella	5.70206125			-
Bacteria.Bacteroidetes.Cytophagia.Cytophagales.Cytophagaceae.Adhaeribacter	6.401017409	NF	6.036251202	0.027323722
Bacteria.Actinobacteria.Actinobacteria.Corynebacteriales.Nocardiaceae.Smaragdicoccus	4.115576002			-
Bacteria.Proteobacteria.Betaproteobacteria.Burkholderiales.Oxalobacteraceae.Oxalicibacterium	5.618435613	NF	5.247882023	0.027323722
Bacteria.Proteobacteria.Gammaproteobacteria.1013_28_CG33	3.497353564			-
Bacteria.Cyanobacteria.Cyanobacteria.SubsectionIII.FamilyI.Planktothrix	3.61956934			-
Bacteria.Proteobacteria.Alphaproteobacteria	5.768597006			-
Bacteria.Proteobacteria.Alphaproteobacteria.Rhodospirillales.Rhodospirillaceae	6.669686982			-
Bacteria.Proteobacteria.Alphaproteobacteria.Rhizobiales.Hyphomicrobiaceae.Hyphomicrobiu	6.244993609			-

m				
Bacteria.Proteobacteria.Gammaproteobacteria.Aeromonadales.Aeromonadaceae.Aeromonas	5.279751993			-
Bacteria.Actinobacteria.Actinobacteria.Euzebyales.Euzebyaceae.Euzebya	4.702670382	NF	4.483802132	0.022109128
Bacteria.Proteobacteria.Alphaproteobacteria.Caulobacterales.Hyphomonadaceae.Woodsholea	5.775641945	NF	5.399417834	0.027323722
Bacteria.Firmicutes.Bacilli.Bacillales.Bacillaceae	6.402443342	F_3	6.069425242	0.027323722
Bacteria.Cyanobacteria.Cyanobacteria.SubsectionIII	5.653292109	NF	5.35482932	0.0265095
Bacteria.Proteobacteria.Betaproteobacteria.Methylophilales.Methylophilaceae	6.208678538			-
Bacteria.Proteobacteria.Deltaproteobacteria.Bdellovibrionales.Bacteriovoracaceae	4.905262848	NF	4.642114082	0.034735259
Bacteria.Armatimonadetes	6.564036374			-
Bacteria.Actinobacteria.Actinobacteria.Frankiales.Geodermatophilaceae.Geodermatophilus	4.665236419			-
Bacteria.Proteobacteria.Betaproteobacteria	5.199572466			-
Bacteria.Proteobacteria.Betaproteobacteria.Burkholderiales.Alcaligenaceae.Oligella	5.467439795	F_3	5.171037393	0.024133897
Bacteria.Lentisphaerae.PBS_III_20	3.647598157			-
Bacteria.Proteobacteria.Epsilonproteobacteria.Campylobacterales.Helicobacteraceae	3.774471411			-
Bacteria.Chloroflexi.Chloroflexia.Kallotenuales.AKIW781	5.39931663			-
Bacteria.Actinobacteria.Actinobacteria.Corynebacteriales	5.381311239	F_3	5.10990745	0.024133897
Bacteria.Proteobacteria.Alphaproteobacteria.Rhodospirillales.Rhodospirillales_Incertae_Sedis .Candidatus_Alysiosphaera	4.381366849			-
Bacteria.Chloroflexi.Chloroflexia.Chloroflexales.Chloroflexaceae	4.127876005			-
Bacteria.Firmicutes.Bacilli.Bacillales.Thermoactinomycetaceae.Hazenella	5.052104985	NF	4.775305311	0.022109128
Bacteria.Proteobacteria.Betaproteobacteria.Neisseriales.Neisseriaceae.Pseudogulbenkiania	3.804662623			-
Bacteria.Proteobacteria.Alphaproteobacteria.Rhizobiales.Xanthobacteraceae.Labrys	4.36890482	NF	5.094306214	0.034863256
Bacteria.Cyanobacteria.Cyanobacteria.SubsectionIV.FamilyI.Nostoc	4.468970046			-
Bacteria.Gemmatimonadetes.Gemmatimonadetes.Gemmatimonadales	7.239067641			-
Bacteria.Proteobacteria.SK259	3.876625464			-

Bacteria.Deinococcus_Thermus.Deinococci.Deinococcales.Deinococcaceae	3.647598157			-
Bacteria.Bacteroidetes.Cytophagia.Cytophagales.Cytophagaceae.Leadbetterella	4.303102378			-
Bacteria.Omnitrophica.NPL_UPA2	5.232740697	F_3	4.99011128	0.03899022
Bacteria.Firmicutes.Clostridia.Clostridiales.Clostridiaceae_3.Brassicibacter	3.61956934			-
Bacteria.Latescibacteria	5.075028325			-
Bacteria.Actinobacteria.Actinobacteria.Streptosporangiales.Nocardiopsaceae	5.184115452	F_1	4.896992275	0.049647804
Bacteria.Proteobacteria.Gammaproteobacteria.Salinisphaerales.Salinisphaeraceae	4.595738803			-
Bacteria.Firmicutes	7.694363737			-
Bacteria.Bacteroidetes.Flavobacteriia.Flavobacteriales.Flavobacteriaceae.Chryseobacterium	5.59326017			-
Bacteria.Proteobacteria.Gammaproteobacteria.Chromatiales.Chromatiaceae	4.72886478			-
Bacteria.Fibrobacteres.Fibrobacteria.Fibrobacterales	5.601197577			-
Bacteria.Lentisphaerae.WCHB1_41	3.860303446			-
Bacteria.Firmicutes.Bacilli.Bacillales.Bacillaceae.Halobacillus	4.951425197			-
Bacteria.Bacteroidetes.Cytophagia.Order_II	5.557004004	F_3	5.189888377	0.03899022
Bacteria.Chloroflexi.S085	5.793945533			-
Bacteria.Actinobacteria.Actinobacteria.Micromonosporales.Micromonosporaceae.Phytomonospora	4.050677799			-
Bacteria.Bacteroidetes.Sphingobacteriia.Sphingobacteriales.Chitinophagaceae.Filimonas	6.191700838	F_3	5.750404737	0.027323722
Bacteria.Firmicutes.Clostridia.Clostridiales.Lachnospiraceae.Lachnoclostridium_5	4.30846588			-
Bacteria.Proteobacteria.Alphaproteobacteria.Rhodospirillales.Rhodospirillaceae.Defluviicoccus	5.714764316			-
Bacteria.Proteobacteria.Alphaproteobacteria.Rickettsiales.Rickettsiales_Incertae_Sedis.Candidatus_Captivus	4.203980638			-
Bacteria.Planctomycetes.Phycisphaerae.Phycisphaerales.Phycisphaeraceae.SM1A02	5.933388205			-
Bacteria.Proteobacteria.Deltaproteobacteria.Desulfobacterales.Desulfobulbaceae.Desulfobulb	3.4892356			-

us				
Bacteria.Chloroflexi.Thermomicrobia.Sphaerobacterales.Sphaerobacteraceae.Nitrolancea	6.520407277	F_3	6.200819071	0.03899022
Bacteria.Firmicutes.Clostridia.Clostridiales.Lachnospiraceae	5.366497301			-
Bacteria.Proteobacteria.Gammaproteobacteria.Alteromonadales	4.597004374			-
Bacteria.Verrucomicrobia.Spartobacteria.Chthoniobacterales.DA101_soil_group	5.028280329			-
Bacteria.Bacteroidetes.Cytophagia.Order_III	4.024348835			-
Bacteria.Proteobacteria.Alphaproteobacteria.Rickettsiales.SM2D12	5.752499598			-
Bacteria.Planctomycetes.Planctomycetacia.Planctomycetales.Planctomycetaceae.Pirellula	5.153540956			-
Bacteria.Actinobacteria.Actinobacteria.Glycomycetales	4.463861735			-
Bacteria.Proteobacteria.Gammaproteobacteria.Chromatiales	5.269550821			-
Bacteria.Bacteroidetes.Flavobacteriia.Flavobacteriales.Flavobacteriaceae.Ulvibacter	5.252455011			-
Bacteria.Proteobacteria.Deltaproteobacteria.Myxococcales.Haliangiaceae	6.474605509			-
Bacteria.Firmicutes.Clostridia.Clostridiales.Family_XI.Tepidimicrobium	4.124938571			-
Bacteria.Proteobacteria.Betaproteobacteria.Burkholderiales.Comamonadaceae.Caenimonas	5.675365359			-
Bacteria.Actinobacteria.Actinobacteria.Micrococcales.Micrococcaceae.Arthrobacter	6.203653012			-
Bacteria.Actinobacteria.Actinobacteria.Frankiales.Frankiaceae	4.390771792	F_3	5.107569246	0.034735259
Bacteria.Firmicutes.Erysipelotrichia.Erysipelotrichales	5.614558326	F_1	5.219151128	0.03899022
Bacteria.Proteobacteria.Alphaproteobacteria.Rhizobiales.Phyllobacteriaceae.Mesorhizobium	6.746183035	F_3	6.353527621	0.027323722
Bacteria.Planctomycetes.Planctomycetacia.Planctomycetales.Planctomycetaceae.Gemmata	4.112102385			-
Bacteria.Actinobacteria.Actinobacteria.Actinomycetales.Actinomycetaceae	5.150049761	F_3	4.888189532	0.033985607
Bacteria.Proteobacteria.Deltaproteobacteria.Myxococcales.Polyangiaceae	4.075501406			-
Bacteria.Chloroflexi.Thermomicrobia.Sphaerobacterales.Sphaerobacteraceae	4.36446601			-
Bacteria.Firmicutes.Bacilli.Bacillales.Staphylococcaceae.Staphylococcus	5.823218748	F_3	5.531020178	0.027323722
Bacteria.Lentisphaerae.Oligosphaeria	4.3352524			-
Bacteria.Actinobacteria.Actinobacteria.Frankiales.Geodermatophilaceae.Blastococcus	5.477076622			-

Bacteria.Proteobacteria.Alphaproteobacteria.Rhodospirillales.Rhodospirillales_Incertae_Sedis.Geminicoccus	4.737972978			-
Bacteria.Proteobacteria.Deltaproteobacteria.Myxococcales.Myxococcaceae	3.774471411			-
Bacteria.Proteobacteria.Alphaproteobacteria.Rhizobiales.Rhizobiales_Incertae_Sedis	7.090161604			-
Bacteria.Proteobacteria.Alphaproteobacteria.Rhodobacterales.Rhodobacteraceae.Roseobacter_clade_CHAB_I_5_lineage	3.774471411			-
Bacteria.Verrucomicrobia.Verrucomicrobia_Incertae_Sedis	4.161333442			-
Bacteria.Bacteroidetes.Cytophagia.Cytophagales.Cytophagaceae.Chryseolinea	5.722139015			-
Bacteria.Bacteroidetes.Cytophagia.Order_II.Rhodothermaceae	5.557004004	F_3	5.215870875	0.03899022
Bacteria.Actinobacteria.Actinobacteria.Frankiales.Cryptosporangiaceae.Fodinicola	3.647598157			-
Bacteria.Proteobacteria.Alphaproteobacteria.Rhodospirillales.MNC12	5.55165511			-
Bacteria.Proteobacteria.Alphaproteobacteria.Rhizobiales.Beijerinckiaceae	5.34107062			-
Bacteria.Firmicutes.Bacilli.Bacillales.Planococcaceae.Lysinibacillus	5.538590271			-
Bacteria.Proteobacteria.Alphaproteobacteria.Rhizobiales.Rhizobiales_Incertae_Sedis.Agaricicola	5.864752731	F_3	5.375494028	0.03899022
Bacteria.Proteobacteria.Alphaproteobacteria.Rhizobiales.Beijerinckiaceae.Camelimonas	4.955232316			-
Bacteria.Planctomycetes.vadinHA49	5.362277094			-
Bacteria.Firmicutes.Bacilli.Lactobacillales.Carnobacteriaceae.Carnobacterium	4.513824474			-
Bacteria.Proteobacteria.Deltaproteobacteria.Myxococcales.P3OB_42	5.271312865			-
Bacteria.Proteobacteria.Betaproteobacteria.Burkholderiales.Alcaligenaceae.Eoetvoesia	4.969666282			-
Bacteria.Actinobacteria.Actinobacteria.Streptosporangiales.Streptosporangiaceae.Nonomuraea	6.330859141	F_3	6.035627524	0.027323722
Bacteria.Bacteroidetes.Sphingobacteriia.Sphingobacteriales.Sphingobacteriaceae.Pedobacter	5.975545234	NF	5.573188841	0.03899022
Bacteria.Cyanobacteria.Cyanobacteria.SubsectionIV.FamilyI.Trichormus	3.61956934			-
Bacteria.Parcubacteria	6.453719331			-

Bacteria.Proteobacteria.Deltaproteobacteria	4.922079954			-
Bacteria.Proteobacteria.Gammaproteobacteria.Thiotrichales.Thiotrichaceae.Methylohalomonas	5.156907683	F_3	4.900642646	0.0265095
Bacteria.Proteobacteria.Gammaproteobacteria.Alteromonadales.Alteromonadaceae.Marinobacter	4.150785129			-
Bacteria.Proteobacteria.Alphaproteobacteria.Rhizobiales.Bradyrhizobiaceae.Bradyrhizobium	6.670935154	F_3	6.314770307	0.027323722
Bacteria.Actinobacteria.Actinobacteria.Pseudonocardiales.Pseudonocardiaceae.Actinophytocola	4.774607187	F_3	4.704329078	0.034735259
Bacteria.Actinobacteria.Actinobacteria.Frankiales.Acidothermaceae	3.876625464			-
Bacteria.Proteobacteria.Deltaproteobacteria.Syntrophobacterales.Syntrophaceae	4.313503427	NF	4.513441608	0.022109128
Bacteria.Proteobacteria.Deltaproteobacteria.Bdellovibrionales.Bdellovibrionaceae	6.224563214			-
Bacteria.Proteobacteria.Gammaproteobacteria.Pseudomonadales.Moraxellaceae.Perlucidibaca	3.804662623			-
Bacteria.Actinobacteria.Actinobacteria.Acidimicrobiales.Iamiaceae.Iamia	5.680546504			-
Bacteria.Actinobacteria.Actinobacteria.Acidimicrobiales.OM1_clade	5.219752632			-
Bacteria.Actinobacteria.Actinobacteria.Micrococcales.Microbacteriaceae.Pseudoclavibacter	4.024348835			-
Bacteria.Proteobacteria.Betaproteobacteria.Nitrosomonadales.Nitrosomonadaceae	6.908779482			-
Bacteria.Chloroflexi.Caldilineae	5.976148521			-
Bacteria.Bacteroidetes.Cytophagia.Cytophagales	4.251811825			-
Bacteria.Chloroflexi.Thermomicrobia.JG30_KF_CM45	6.359021968			-
Bacteria.Bacteroidetes.Sphingobacteriia.Sphingobacteriales.Chitinophagaceae.Parasegetibacter	5.364689802			-
Bacteria.Proteobacteria.Alphaproteobacteria.Caulobacterales	6.783309826			-
Bacteria.Bacteroidetes.Cytophagia.Cytophagales.Cytophagaceae	7.284410546			-
Bacteria.Bacteroidetes.Sphingobacteriia.Sphingobacteriales.Saprospiraceae.Aureispira	4.319369978			-

Bacteria.Proteobacteria.Alphaproteobacteria.Rhizobiales.Rhizobiales_Incertae_Sedis.Nordella	6.447428232			-
Bacteria.Acidobacteria.Acidobacteria.Subgroup_10.CA002	5.08085375	NF	4.802841755	0.024133897
Bacteria.Proteobacteria.Betaproteobacteria.Burkholderiales.Alcaligenaceae.Castellaniella	5.625132569			-
Bacteria.Proteobacteria.Alphaproteobacteria.Rhizobiales.Bradyrhizobiaceae.Bosea	5.919587429			-
Bacteria.Verrucomicrobia.Spartobacteria.Chthoniobacterales.01D2Z36	4.398412896	NF	4.40822269	0.022109128
Bacteria.Proteobacteria.Betaproteobacteria.Burkholderiales.Comamonadaceae.Rhizobacter	4.169571125	NF	4.830886018	0.022109128
Bacteria.Bacteroidetes.Sphingobacteriia.Sphingobacteriales.LiUU_11_161	4.067874824			-
Bacteria.Proteobacteria.Gammaproteobacteria.Cellvibrionales.Cellvibrionaceae.Simiduia	5.214199234			-
Bacteria.Proteobacteria.Deltaproteobacteria.Myxococcales.Blfdi19	5.702680422			-
Bacteria.Firmicutes.Bacilli.Bacillales.Staphylococcaceae	6.485635006			-
Bacteria.Actinobacteria.Actinobacteria.Acidimicrobiales	6.353823655			-
Bacteria.Firmicutes.Clostridia.Clostridiales.Clostridiaceae_1.Clostridium_sensu_stricto_10	4.653739747	F_1	4.59735013	0.034735259
Bacteria.Firmicutes.Clostridia.Clostridiales.Clostridiaceae_1.Clostridium_sensu_stricto_13	5.402732529			-
Bacteria.Firmicutes.Clostridia.Clostridiales.Clostridiaceae_1.Clostridium_sensu_stricto_12	4.818175156			-
Bacteria.Firmicutes.Clostridia.Clostridiales.Clostridiaceae_1.Clostridium_sensu_stricto_15	3.497353564			-
Bacteria.Proteobacteria.Betaproteobacteria.Methylophilales.Methylophilaceae.Methylotenera	5.42096371	NF	5.089529939	0.027323722
Bacteria.Proteobacteria.Alphaproteobacteria.Parvularculales.Parvularculaceae	4.372633922	F_1	4.564759654	0.045767773
Bacteria.Proteobacteria.Deltaproteobacteria.Oligoflexales	5.597410871			-
Bacteria.Proteobacteria.Betaproteobacteria.Rhodocyclales	5.057237472			-
Bacteria.Proteobacteria.Alphaproteobacteria.Rickettsiales.RB446	5.119301479			-
Bacteria.Proteobacteria.Deltaproteobacteria.Desulfobacterales	4.803260496	NF	4.539999322	0.0265095
Bacteria.Proteobacteria.Gammaproteobacteria.Xanthomonadales.Xanthomonadaceae.Rhodanobacter	6.675239898	F_3	6.357663985	0.027323722
Bacteria.Proteobacteria.Betaproteobacteria.Nitrosomonadales.Nitrosomonadaceae.Nitrosospira	5.798080415			-

Bacteria.Gemmatimonadetes.Gemmatimonadetes.BD2_11_terrestrial_group	5.662320542			-
Bacteria.Bacteroidetes.Sphingobacteriia.Sphingobacteriales.Chitinophagaceae.Flavihumibacter	4.429906054			-
Bacteria.Proteobacteria.Alphaproteobacteria.Rhizobiales.BCf3_20	5.668524369			-
Bacteria.TA06	5.639966078			-
Bacteria.SM2F11	5.842139058			-
Bacteria.Proteobacteria.Gammaproteobacteria	5.958116754			-
Bacteria.Actinobacteria.Actinobacteria	5.803048031	NF	5.449453277	0.027323722
Bacteria.Proteobacteria.Deltaproteobacteria.Myxococcales.Cystobacteraceae	5.240411759			-
Bacteria.Firmicutes.Bacilli.Lactobacillales.Aerococcaceae.Aerosphaera	5.35633281			-
Bacteria.Proteobacteria.Betaproteobacteria.Burkholderiales.Alcaligenaceae.Pusillimonas	5.633495214			-
Bacteria.Bacteroidetes.Flavobacteriia.Flavobacteriales.Flavobacteriaceae.Tamlana	5.35116014			-
Bacteria.Bacteroidetes.Flavobacteriia.Flavobacteriales.Cryomorphaceae.Wandonia	3.4892356			-
Bacteria.Proteobacteria.Alphaproteobacteria.Rhodospirillales.Rhodospirillaceae.Dongia	6.303750191	F_3	5.889427059	0.027323722
Bacteria.Proteobacteria.Gammaproteobacteria.Enterobacteriales.Enterobacteriaceae.Citrobacter	5.232059216			-
Bacteria.Acidobacteria	7.563792629			-
Bacteria.Proteobacteria.Gammaproteobacteria.Thiotrichales.Piscirickettsiaceae.Methylophaga	4.747818359			-
Bacteria.Proteobacteria.Deltaproteobacteria.Myxococcales.Vulgatibacteraceae	4.422799542			-
Bacteria.Chloroflexi.Chloroflexia.Chloroflexales.FFCH7168	4.689730696	NF	4.409949315	0.034735259
Bacteria.Firmicutes.Clostridia.Clostridiales.Peptostreptococcaceae.Sporacetigenium	4.439120194			-
Bacteria.Actinobacteria.Actinobacteria.Acidimicrobiales.Acidimicrobiales_Incertae_Sedis	5.628118468	F_3	5.298892499	0.027323722
Bacteria.Bacteroidetes.Cytophagia.Cytophagales.Flammeovirgaceae.Luteivirga	4.105692619			-
Bacteria.Proteobacteria.Alphaproteobacteria.Rhodospirillales.Rhodospirillaceae.Thalassobaculum	4.200659254			-

Bacteria.Proteobacteria.Betaproteobacteria.Hydrogenophilales.Hydrogenophilaceae	3.885180936			-
Bacteria.Actinobacteria.Actinobacteria.Micromonosporales.Micromonosporaceae.Dactylosporangium	4.81883184	NF	4.546540591	0.034735259
Bacteria.Spirochaetae.Spirochaetes	5.392424004			-
Bacteria.Actinobacteria.Actinobacteria.Micrococcales.Intrasporangiaceae	5.19811822			-
Bacteria.Proteobacteria.Gammaproteobacteria.Oceanospirillales.Oceanospirillaceae	6.106589158	NF	5.707905811	0.03899022
Bacteria.Firmicutes.Bacilli.Bacillales.Staphylococcaceae.Aliicoccus	5.304567664			-
Bacteria.TM6	6.398238638			-
Bacteria.Proteobacteria.Deltaproteobacteria.GR_WP33_30	6.282364724	NF	5.891893382	0.027323722
Bacteria.Bacteroidetes.Flavobacteriia.Flavobacteriales.Flavobacteriaceae.Aequorivita	5.155514557			-
Bacteria.Chlamydiae.Chlamydiae.Chlamydiales.cvE6	6.275445453	F_3	5.958516155	0.03899022
Bacteria.Actinobacteria.Actinobacteria.Micromonosporales.Micromonosporaceae.Actinoplanes	5.013561864			-
Bacteria.Proteobacteria.Deltaproteobacteria.Myxococcales.KD3_10	5.418055561	NF	5.104385768	0.027323722
Bacteria.Actinobacteria.Actinobacteria.Streptomycetales.Streptomycetaceae	6.166213909			-
Bacteria.Actinobacteria.Actinobacteria.Micrococcales.Promicromonosporaceae.Isoptericola	5.265429087			-
Bacteria.Firmicutes.Bacilli.Bacillales.Bacillaceae.Terribacillus	4.337643861			-
Bacteria.Proteobacteria.Gammaproteobacteria.Xanthomonadales.Xanthomonadaceae.Thermomonas	6.208727037			-
Bacteria.Proteobacteria.Gammaproteobacteria.Xanthomonadales.Nevskiaceae	4.639932201			-
Bacteria.Proteobacteria.Alphaproteobacteria.Rickettsiales.AKIW1012	4.966575964			-
Bacteria.Actinobacteria.Actinobacteria.Propionibacteriales.Propionibacteriaceae	4.486860167			-
Bacteria.Proteobacteria.Deltaproteobacteria.Oligoflexales.Oligoflexaceae	5.292033417			-
Bacteria.Actinobacteria.Actinobacteria.Micrococcales.Ruaniaceae.Ruania	4.144977042			-
Bacteria.Proteobacteria.Deltaproteobacteria.Myxococcales.27F_1492R	4.823103345	NF	4.659841514	0.024133897

Bacteria.Fibrobacteres.Fibrobacteria.Fibrobacterales.Fibrobacteraceae	5.354981993	F_3	5.065524327	0.027323722
Bacteria.Actinobacteria.Actinobacteria.Propionibacteriales.Nocardioidaceae.Marmoricola	5.69998755			-
Bacteria.Actinobacteria.Actinobacteria.Micrococcales.Microbacteriaceae.Leucobacter	6.404055008	F_3	6.029147509	0.027323722
Bacteria.Actinobacteria.Actinobacteria.Micrococcales.Dermabacteraceae	6.033381236			-
Bacteria.Firmicutes.Bacilli.Lactobacillales.Carnobacteriaceae.Atopostipes	5.353202821	F_3	5.042958426	0.027323722
Bacteria.Actinobacteria.Actinobacteria.Micromonosporales.Micromonosporaceae	6.348387294			-
Bacteria.Actinobacteria.Actinobacteria.Pseudonocardiales.Pseudonocardiaceae.Amycolatopsis	5.329112495	F_3	5.076403517	0.033985607
Bacteria.Bacteroidetes.Flavobacteriia.Flavobacteriales.Flavobacteriaceae.Croceibacter	3.869446796			-
Bacteria.Chlorobi.Chlorobia	5.979792789			-
Bacteria.Proteobacteria.Betaproteobacteria.Burkholderiales.Alcaligenaceae.Parapusillimonas	3.4892356			-
Bacteria.Chloroflexi.Ardenticatenia	4.547475685			-
Bacteria.Actinobacteria.Actinobacteria.Solirubrobacterales	4.645962689	NF	4.957846589	0.033985607
Bacteria.Actinobacteria.Actinobacteria.Pseudonocardiales.Pseudonocardiaceae	5.382264989	F_3	5.089704438	0.049647804
Bacteria.Proteobacteria.Alphaproteobacteria.Sphingomonadales.WW2_159	5.923032852			-
Bacteria.Cyanobacteria	6.293990014			-
Bacteria.Proteobacteria.Alphaproteobacteria.Rhodospirillales.Rhodospirillaceae.Azospirillum	4.186210932			-
Bacteria.Proteobacteria.Alphaproteobacteria.Rhizobiales.Hyphomicrobiaceae.Filomicrobium	4.425968567			-
Bacteria.Actinobacteria.Actinobacteria.Micrococcales.Ruaniaceae	4.144977042			-
Bacteria.Proteobacteria.Deltaproteobacteria.Bdellovibrionales.Bdellovibrionaceae.Bdellovibrio	6.050846028			-
Bacteria.Fibrobacteres	5.601197577			-
Bacteria.Bacteroidetes.Cytophagia.Cytophagales.Cytophagaceae.Nibribacter	5.215203469	NF	5.060178158	0.022109128
Bacteria.Verrucomicrobia.Verrucomicrobiae.Verrucomicrobiales.Verrucomicrobiaceae.Verrucomicrobium	4.599009915			-
Bacteria.Spirochaetae.Spirochaetes.Spirochaetales.Leptospiraceae.Turneriella	5.087369429	NF	4.791573256	0.033985607

Bacteria.Acidobacteria.Acidobacteria.Acidobacteriales	6.059742316			-
Bacteria.Proteobacteria.Gammaproteobacteria.Alteromonadales.Alteromonadaceae	4.150785129			-
Bacteria.Proteobacteria.Alphaproteobacteria.Rhizobiales.Hyphomicrobiaceae.Rhodomicrobium	4.687259019	NF	4.433279296	0.022109128
Bacteria.Firmicutes.Clostridia.Clostridiales.Peptostreptococcaceae	4.218168878			-
Bacteria.Proteobacteria.Deltaproteobacteria.Myxococcales.Vulgatibacteraceae.Vulgatibacter	4.422799542			-
Bacteria.Proteobacteria.Gammaproteobacteria.Chromatiales.Ectothiorhodospiraceae.Acidiferrobacter	5.259053714			-
Bacteria.Actinobacteria.Actinobacteria.Kineosporiales.Kineosporiaceae	5.476929787	F_3	5.169723741	0.03899022
Bacteria.Bacteroidetes.Sphingobacteriia.Sphingobacteriales.Sphingobacteriaceae.Solitalea	5.405171147			-
Bacteria.Bacteroidetes.Flavobacteriia.Flavobacteriales.Flavobacteriaceae.Moheibacter	5.403873888			-
Bacteria.Firmicutes.OPB54	4.744434469			-
Bacteria.Bacteroidetes.Cytophagia.Cytophagales.Flammeovirgaceae.Marinoscillum	4.88390677	NF	4.800874742	0.022109128
Bacteria.Proteobacteria.Alphaproteobacteria.Sphingomonadales.Sphingomonadaceae	5.935992223	F_3	5.545473457	0.027323722
Bacteria.Actinobacteria.Actinobacteria.Micrococcales.Demequinaceae	5.045956383			-
Bacteria.Proteobacteria.Alphaproteobacteria.Rhodospirillales.Rhodospirillaceae.Ferrovibrio	5.107353519			-
Bacteria.Chloroflexi.Ktedonobacteria.Ktedonobacterales	5.142253533	F_3	5.28757673	0.022109128
Bacteria.Actinobacteria.Actinobacteria.Micromonosporales.Micromonosporaceae.Rhizocola	4.348464869	NF	4.881393648	0.022109128
Bacteria.Chlamydiae	6.71018454	F_3	6.314596974	0.027323722
Bacteria.Proteobacteria.Alphaproteobacteria.Rickettsiales.Rickettsiales_Incertae_Sedis.Constrictibacter	4.517591092			-
Bacteria.Proteobacteria.Gammaproteobacteria.Order_Incertae_Sedis	5.810659761			-
Bacteria.Bacteroidetes.Flavobacteriia	6.526539391			-
Bacteria.Proteobacteria.Alphaproteobacteria.Rhodospirillales.Rhodospirillaceae.Inquilinus	4.807199239			-
Bacteria.Proteobacteria.Betaproteobacteria.Nitrosomonadales.Nitrosomonadaceae.Nitrosomo	5.871568619			-

nas				
Bacteria.Firmicutes.Bacilli.Lactobacillales.Aerococcaceae.Facklamia	5.904738419	F_3	5.588905778	0.027323722
Bacteria.Actinobacteria.Actinobacteria.Micromonosporales	6.702242585			-
Bacteria.Actinobacteria.Actinobacteria.Propionibacteriales	6.479236126			-
Bacteria.Bacteroidetes.Flavobacteriia.Flavobacteriales.Flavobacteriaceae.Flavobacterium	6.324303314	F_1	5.924519619	0.03899022
Bacteria.Proteobacteria.Alphaproteobacteria.Sphingomonadales.DSSF69	5.502407922			-
Bacteria.Proteobacteria.Gammaproteobacteria.Enterobacteriales.Enterobacteriaceae	5.448485961			-
Bacteria.Actinobacteria.Actinobacteria.Pseudonocardiales.Pseudonocardiaceae.Lentzea	4.075501406			-
Bacteria.Proteobacteria.Betaproteobacteria.Rhodocyclales.Rhodocyclaceae.Georgfuchsia	4.879540195			-
Bacteria.Actinobacteria.Actinobacteria.Micromonosporales.Micromonosporaceae.Stackebrandtia	3.804662623			-
Bacteria.Bacteroidetes.Bacteroidia.Bacteroidales	5.797201593			-
Bacteria.Proteobacteria.Alphaproteobacteria.Sphingomonadales.SWB04	4.784408887	F_1	4.592351815	0.022109128
Bacteria.Proteobacteria.Betaproteobacteria.B1_7BS	5.222881632			-
Bacteria.Chlamydiae.Chlamydiae.Chlamydiales.Parachlamydiaceae.Candidatus_Protochlamydia	4.014280396			-
Bacteria.Proteobacteria.Alphaproteobacteria.Rhizobiales.Hyphomicrobiaceae.Pedomicrobium	5.976777219			-
Bacteria.Actinobacteria.Actinobacteria.Solirubrobacterales.Solirubrobacteraceae	4.466688158			-
Bacteria.Actinobacteria.Actinobacteria.Corynebacteriales.Corynebacteriaceae	6.959085168	F_3	6.614863701	0.027323722
Bacteria.Planctomycetes.Planctomycetacia.Planctomycetales.Planctomycetaceae.Pir4_lineage	4.484998931			-
Bacteria.Chloroflexi.JG30_KF_CM66	5.589411645	NF	4.958304918	0.03899022
Bacteria.Chlorobi.Chlorobia.Chlorobiales	5.979792789			-
Bacteria.Chlamydiae.Chlamydiae.Chlamydiales.Waddliaceae.Waddlia	5.548152102	F_3	5.26670183	0.024133897
Bacteria.Actinobacteria.Actinobacteria.Corynebacteriales.Corynebacteriaceae.Corynebacterium	4.88335611			-

Bacteria.Bacteroidetes.Bacteroidia.Bacteroidales.Marinilabiaceae	5.249726723	NF	4.929771099	0.037941542
Bacteria.Firmicutes.Bacilli.Bacillales.Thermoactinomycetaceae.Thermoactinomyces	4.58844312			-
Bacteria.Bacteroidetes.Cytophagia.Cytophagales.Flammeovirgaceae.Candidatus_Amoebophilus	4.075501406			-
Bacteria.Aerophobetes	4.612605427			-
Bacteria.Chloroflexi.Chloroflexia.Herpetosiphonales	5.240403989			-
Bacteria.Proteobacteria.Alphaproteobacteria.Rhizobiales.Bradyrhizobiaceae.Rhodopseudomonas	5.224939944			-
Bacteria.Candidate_division_WS6	5.755047677			-
Bacteria.Actinobacteria.Actinobacteria.Frankiales.Nakamurellaceae.Nakamurella	4.650284467			-
Bacteria.Proteobacteria.Gammaproteobacteria.Xanthomonadales.Solimonadaceae.Fontimonas	4.649582571			-
Bacteria.Firmicutes.Clostridia.Clostridiales.Peptococcaceae	4.595475082			-
Bacteria.Proteobacteria.Gammaproteobacteria.Legionellales	4.766800122			-
Bacteria.Chloroflexi.KD4_96	6.620546415	F_3	6.156850567	0.027323722
Bacteria.Firmicutes.Bacilli.Lactobacillales.Enterococcaceae	5.707840379	F_3	5.310529425	0.027323722
Bacteria.Actinobacteria.Actinobacteria.Bifidobacteriales	4.69780032			-
Bacteria.Actinobacteria.Actinobacteria.Streptosporangiales.Thermomonosporaceae.Actinomadura	5.929351371	F_3	5.596262544	0.027323722
Bacteria.Verrucomicrobia.Spartobacteria.Chthoniobacterales.LD29	4.390771792			-
Bacteria.Actinobacteria.Actinobacteria.Micrococcales	5.602347114			-
Bacteria.Proteobacteria.Alphaproteobacteria.Caulobacterales.Caulobacteraceae.Phenylobacterium	6.283654171	F_3	5.83466073	0.03899022
Bacteria.Actinobacteria.Actinobacteria.Propionibacteriales.Nocardioidaceae.Nocardioides	6.069695462			-
Bacteria.JL_ETNP_Z39	5.568817202	NF	5.27049971	0.033985607
Bacteria.Proteobacteria.Epsilonproteobacteria.Campylobacterales	4.892283427			-

Bacteria.Proteobacteria.Gammaproteobacteria.Oceanospirillales.Halomonadaceae.Halomonas	5.1257995	F_3	4.868865409	0.027323722
Bacteria.Proteobacteria.Alphaproteobacteria.Rhodospirillales.JG37_AG_20	6.199283406			-
Bacteria.Proteobacteria.Alphaproteobacteria.Parvularculales.Parvularculaceae.Parvularcula	4.372633922	F_1	4.543491056	0.045767773
Bacteria.Microgenomates	4.652957984			-
Bacteria.Proteobacteria.JTB23	4.235869818			-
Bacteria.Proteobacteria.Deltaproteobacteria.Myxococcales.Haliangiaceae.Haliangium	6.474605509			-
Bacteria.Proteobacteria.Gammaproteobacteria.Legionellales.Legionellaceae	5.87582646	F_3	5.520206543	0.03899022
Bacteria.Verrucomicrobia.Spartobacteria.Chthoniobacterales.Chthoniobacteraceae.Chthoniobacter	5.984318909			-
Bacteria.Firmicutes.Clostridia.Clostridiales.Clostridiaceae_2.Alkaliphilus	4.505074195			-
Bacteria.Firmicutes.Bacilli	7.673112536			-
Bacteria.Actinobacteria.Actinobacteria.Euzebyales.Euzebyaceae	4.702670382	NF	4.464529326	0.022109128
Bacteria.Firmicutes.Bacilli.Bacillales.Staphylococcaceae.Jeotgalicoccus	6.325950872			-
Bacteria.Proteobacteria.Gammaproteobacteria.Xanthomonadales.Solimonadaceae.Solimonas	4.186210932			-
Bacteria.Proteobacteria.Gammaproteobacteria.Cellvibrionales.Halieaceae	5.886102467			-
Bacteria.Proteobacteria.Deltaproteobacteria.Desulfuromonadales	4.339042015			-
Bacteria.Proteobacteria.Betaproteobacteria.Burkholderiales.Alcaligenaceae.Candidimonas	5.374331134	F_3	5.073376691	0.03899022
Bacteria.Actinobacteria.Actinobacteria.Frankiales.Cryptosporangiaceae	3.647598157			-
Bacteria.Bacteroidetes.Sphingobacteriia.Sphingobacteriales.Chitinophagaceae.Taibaiella	5.804885472			-
Bacteria.Cyanobacteria.Cyanobacteria.SubsectionIII.FamilyI.Phormidium	3.4892356			-
Bacteria.Firmicutes.Bacilli.Bacillales.Bacillaceae.Gracilibacillus	5.06149633	F_3	4.850599878	0.03899022
Bacteria.Proteobacteria.Alphaproteobacteria.Rhizobiales.Rhizobiaceae.Rhizobium	5.943973202			-
Bacteria.Proteobacteria.Alphaproteobacteria.Sphingomonadales	6.65392681	F_3	6.300995584	0.027323722
Bacteria.Cyanobacteria.Cyanobacteria.SubsectionI.FamilyI	4.482418354			-

Bacteria.Actinobacteria.Actinobacteria.Acidimicrobiales.Acidimicrobiaceae.CL500_29_marine_group	5.451065981			-
Bacteria.Proteobacteria.Alphaproteobacteria.Sphingomonadales.Sphingomonadaceae.Novosphingobium	5.960327325			-
Bacteria.Proteobacteria.Gammaproteobacteria.Xanthomonadales.Xanthomonadaceae.Tahibacter	5.07184312			-
Bacteria.Spirochaetae	5.392424004			-
Bacteria.Proteobacteria.Deltaproteobacteria.Myxococcales	5.106350522			-
Bacteria.Elusimicrobia.Elusimicrobia	4.404742487	NF	4.325094019	0.022109128
Bacteria.Proteobacteria.Gammaproteobacteria.Xanthomonadales.Xanthomonadales_Incertae_Sedis	5.612751182			-
Bacteria.Firmicutes.Clostridia.Clostridiales.Ruminococcaceae.Ruminiclostridium_1	4.635251664			-
Bacteria.Actinobacteria.Actinobacteria.Streptosporangiales.Streptosporangiaceae.Microbispora	6.022336679			-
Bacteria.Proteobacteria.Gammaproteobacteria.Oceanospirillales.BPS_CK174	4.611376911	F_3	4.75054536	0.022109128
Bacteria.Proteobacteria.Betaproteobacteria.Burkholderiales.Burkholderiaceae.Burkholderia	5.122778876			-
Bacteria.Proteobacteria.Betaproteobacteria.Burkholderiales.Comamonadaceae.Ramlibacter	6.019599761			-
Bacteria.Chloroflexi.Ktedonobacteria.Ktedonobacterales.Thermosporotrichaceae	5.142253533	F_3	4.949424432	0.022109128
Bacteria.Chloroflexi.Thermomicrobia	6.850683356	F_3	6.40990622	0.027323722
Bacteria.Actinobacteria.Actinobacteria.Gaiellales.Gaiellaceae.Gaiella	6.0515098	NF	5.714608026	0.027323722
Bacteria.Proteobacteria.Gammaproteobacteria.PYR10d3	4.418054718	F_1	4.489933197	0.022109128
Bacteria.Acidobacteria.Acidobacteria.Subgroup_21	4.161333442			-
Bacteria.Firmicutes.Clostridia.Clostridiales.Eubacteriaceae.Alkalibaculum	4.30746151			-
Bacteria.Acidobacteria.Acidobacteria.Subgroup_25	5.326285109	NF	5.050681411	0.033985607
Bacteria.Verrucomicrobia.Verrucomicrobiae.Verrucomicrobiales.Verrucomicrobiaceae	6.042804103			-

Bacteria.Proteobacteria.Gammaproteobacteria.Oceanospirillales.Hahellaceae	3.723318839			-
Bacteria.Chloroflexi.Caldilineae.Caldilineales.Caldilineaceae.Litorilinea	4.02579407			-
Bacteria.Chloroflexi.Caldilineae.Caldilineales.Caldilineaceae	5.976148521			-
Bacteria.Gemmatimonadetes.Gemmatimonadetes.Gemmatimonadales.Gemmatimonadaceae.Gemmatimonas	6.848628654	F_1	6.456635234	0.027323722
Bacteria.Elusimicrobia.Elusimicrobia.Lineage_IV	5.009807687	NF	4.832416793	0.0265095
Bacteria.Proteobacteria.Alphaproteobacteria.Rhizobiales.AKYG1088	4.402447142			-
Bacteria.Proteobacteria.Alphaproteobacteria.Rhizobiales.Hyphomicrobiaceae.Prosthecomicrobium	3.647598157			-
Bacteria.Chlorobi.Ignavibacteria.Ignavibacteriales.BSV26	5.575625658	F_1	5.272837674	0.049647804
Bacteria.Acidobacteria.Acidobacteria.Subgroup_3.Elev_16S_1166	4.914662762			-
Bacteria.Chloroflexi.Ktedonobacteria.C0119	5.201346522			-
Bacteria.Acidobacteria.Acidobacteria.Subgroup_4.RB41	5.990829229	NF	5.693536999	0.027323722
Bacteria.Actinobacteria.Actinobacteria.Micromonosporales.Micromonosporaceae.Luedemannella	5.854916199	F_3	5.507418353	0.03899022
Bacteria.Proteobacteria.Deltaproteobacteria.Myxococcales.Sandaracinaceae.Sandaracinus	5.370839697			-
Bacteria.Planctomycetes.Planctomycetacia.Planctomycetales.Planctomycetaceae	5.942991713			-
Bacteria.Gemmatimonadetes.Gemmatimonadetes.Gemmatimonadales.Gemmatimonadaceae	7.123722042			-
Bacteria.Actinobacteria.Actinobacteria.Micrococcales.Dermabacteraceae.Brachybacterium	6.033381236			-
Bacteria.Proteobacteria.Alphaproteobacteria.Caulobacterales.Hyphomonadaceae	5.964399314			-
Bacteria.Firmicutes.Bacilli.Lactobacillales.Streptococcaceae.Lactococcus	6.361852688			-
Bacteria.Proteobacteria.Alphaproteobacteria.Rhodospirillales.Rhodospirillaceae.Tistlia	3.61956934			-
Bacteria.Actinobacteria.Actinobacteria.Solirubrobacterales.Solirubrobacteraceae.Solirubrobacter	4.466688158			-
Bacteria.Proteobacteria.Betaproteobacteria.Burkholderiales.Burkholderiaceae.Lautropia	5.084289681	NF	4.74927899	0.037941542

Bacteria.Bacteroidetes.Cytophagia.Cytophagales.Cytophagaceae.Rufibacter	5.09529819	NF	4.799443354	0.022109128
Bacteria.Actinobacteria.Actinobacteria.Solirubrobacterales.288_2	5.059530364	NF	4.759122444	0.0265095
Bacteria.Chlorobi.Chlorobia.Chlorobiales.SJA_28	5.433295599	NF	5.147016603	0.022109128
Bacteria.Proteobacteria.Alphaproteobacteria.Rhodobacterales.Rhodobacteraceae.Roseovarius	4.564914708	NF	4.592034033	0.034735259
Bacteria.Proteobacteria.Epsilonproteobacteria.Campylobacterales.Helicobacteraceae.Sulfurimonas	4.874428783			-
Bacteria.Actinobacteria.Actinobacteria.Propionibacteriales.Nocardioidaceae.Kribbella	4.87275813	F_3	5.005630247	0.024133897
Bacteria.Proteobacteria.Gammaproteobacteria.Xanthomonadales.Xanthomonadaceae.Silanimonas	3.647598157			-
Bacteria.Chloroflexi.Chloroflexia.Herpetosiphonales.Herpetosiphonaceae	5.240403989			-
Bacteria.Bacteroidetes.Sphingobacteriia.Sphingobacteriales.PHOS_HE51	5.83090933	NF	5.524813197	0.034863256
Bacteria.Bacteroidetes.Sphingobacteriia.Sphingobacteriales.Chitinophagaceae.Sediminibacterium	5.261232584			-
Bacteria.Planctomycetes.Pla4_lineage	4.686077195	F_3	4.720783883	0.037941542
Bacteria.Proteobacteria	6.244368762			-
Bacteria.Planctomycetes.Pla3_lineage	4.17765546			-
Bacteria.Acidobacteria.Acidobacteria.Holophagales.Holophagaceae.Geothrix	4.791724249	F_3	4.756174725	0.045938131
Bacteria.Proteobacteria.Betaproteobacteria.Burkholderiales.Alcaligenaceae.Bordetella	5.749048893			-
Bacteria.Bacteroidetes.Cytophagia.Cytophagales.Cytophagaceae.Pontibacter	6.162844587	NF	5.780786689	0.027323722
Bacteria.Firmicutes.Bacilli.Bacillales.Thermoactinomycetaceae.Laceyella	3.948628152			-
Bacteria.Proteobacteria.Alphaproteobacteria.Rhizobiales.JG34_KF_361	5.233199997			-
Bacteria.Chlorobi.Ignavibacteria	5.575625658	F_1	5.286544041	0.049647804
Bacteria.Bacteroidetes.Cytophagia	7.418701152			-
Bacteria.Bacteroidetes.Cytophagia.Cytophagales.Flammeovirgaceae.Fulvivirga	3.647598157			-
Bacteria.Elusimicrobia.Elusimicrobia.MVP_88	5.198215885			-

Bacteria.Proteobacteria.Gammaproteobacteria.Salinisphaerales	4.595738803			-
Bacteria.Proteobacteria.Alphaproteobacteria.Rhizobiales.Rhizobiaceae.Kaistia	6.020258204			-
Bacteria.Proteobacteria.Alphaproteobacteria.Caulobacterales.Caulobacteraceae.Asticcacaulis	5.232404383	F_3	4.981446758	0.045938131
Bacteria.Proteobacteria.Gammaproteobacteria.Xanthomonadales.Solimonadaceae	5.037931236			-
Bacteria.Deinococcus_Thermus.Deinococci.Deinococcales.Trueperaceae.Truepera	5.827683817			-
Bacteria.Proteobacteria.Alphaproteobacteria.Caulobacterales.Caulobacteraceae.Brevundimonas	5.971818114			-
Bacteria.Bacteroidetes.Bacteroidia.Bacteroidales.Bacteroidaceae	3.948628152			-
Bacteria.Chloroflexi.Chloroflexia.Chloroflexales.Roseiflexaceae	6.305999296			-
Bacteria.Bacteroidetes.Flavobacteriia.Flavobacteriales.Cryomorphaceae	4.28610286			-
Bacteria.Proteobacteria.Deltaproteobacteria.Myxococcales.Polyangiaceae.Sorangium	5.674875723			-
Bacteria.Acidobacteria.Acidobacteria.Holophagales.Holophagaceae.Holophaga	4.842440127			-
Bacteria.Elusimicrobia	5.958714067			-
Bacteria.Chloroflexi.Chloroflexia	6.390756608			-
Bacteria.Proteobacteria.Alphaproteobacteria.Rhodospirillales.Rhodospirillaceae.Thalassospira	3.934839823			-
Bacteria.Cyanobacteria.Cyanobacteria.SubsectionIII.FamilyI	5.292091884	NF	5.01263401	0.033985607
Bacteria.Spirochaetae.Spirochaetes.Spirochaetales	5.392424004			-
Bacteria.Proteobacteria.Alphaproteobacteria.Rhodospirillales	5.848123907	F_3	5.399411962	0.03899022
Bacteria.Verrucomicrobia.Verrucomicrobiae.Verrucomicrobiales.Verrucomicrobiaceae.Luteolibacter	5.834102556			-
Bacteria.Proteobacteria.Alphaproteobacteria.Rhodospirillales.Rhodospirillales_Incertae_Sedis.Reyranella	6.402625262			-
Bacteria.Proteobacteria.Gammaproteobacteria.Cellvibrionales.Cellvibrionaceae.Marinimicrobium	5.658793531	F_3	5.371643103	0.045938131

Bacteria.Proteobacteria.Gammaproteobacteria.Xanthomonadales.Solimonadaceae.Polycyclovorans	6.309781268	NF	5.996809679	0.027323722
Bacteria.Chlamydiae.Chlamydiae.Chlamydiales.Parachlamydiaceae	4.024348835			-
Bacteria.Verrucomicrobia.Opitutae.Opitutales.Opitutaceae.Opitutus	6.683140002	F_3	6.253430476	0.027323722
Bacteria.Firmicutes.Bacilli.Bacillales.Alicyclobacillaceae	6.046877928			-
Bacteria.Planctomycetes.Planctomycetacia.Planctomycetales	6.522703503	F_3	6.124211704	0.027323722
Bacteria.Gemmatimonadetes	7.284453568			-
Bacteria.Cyanobacteria.Cyanobacteria.SubsectionIII.FamilyI.Microcoleus	4.450301249	NF	4.271298612	0.022109128
Bacteria.Lentisphaerae	4.4163402			-
Bacteria.Proteobacteria.Gammaproteobacteria.Xanthomonadales.Xanthomonadaceae.Mizugakiibacter	6.378652627	F_3	6.070842417	0.027323722
Bacteria.Planctomycetes.Phycisphaerae.Phycisphaerales.Phycisphaeraceae.Phycisphaera	4.595738803			-
Bacteria.Actinobacteria.Actinobacteria.Propionibacteriales.Nocardioidaceae	4.785119466			-
Bacteria.Proteobacteria.Deltaproteobacteria.Myxococcales.BIrii41	6.161447258			-
Bacteria.Verrucomicrobia.Opitutae.Opitutales	6.683140002	F_3	6.231230725	0.027323722
Bacteria.Bacteroidetes.Sphingobacteriia.Sphingobacteriales.Chitinophagaceae.Niabella	5.528188748	F_3	5.213176955	0.03899022
Bacteria.Bacteroidetes.Flavobacteriia.Flavobacteriales	6.526539391			-
Bacteria.Proteobacteria.Gammaproteobacteria.Chromatiales.Ectothiorhodospiraceae.Thioalkalispira	3.5098222			-
Bacteria.Proteobacteria.Alphaproteobacteria.Parvularculales	4.372633922	F_1	4.702345853	0.045767773
Bacteria.Bacteroidetes.Cytophagia.Cytophagales.Flammeovirgaceae	3.497353564			-
Bacteria.Firmicutes.Bacilli.Bacillales	5.317174875			-
Bacteria.Proteobacteria.Alphaproteobacteria.Rhodobacterales.Rhodobacteraceae.Paracocccus	5.476842503			-
Bacteria.Proteobacteria.Betaproteobacteria.Burkholderiales.Alcaligenaceae.Achromobacter	6.05743869			-
Bacteria.Firmicutes.Bacilli.Lactobacillales.Aerococcaceae.Aerococcus	6.083752783	F_3	5.799770607	0.03899022

Bacteria.Acidobacteria.Acidobacteria.Subgroup_3.AMGG11	4.900832166			-
Bacteria.Firmicutes.Bacilli.Bacillales.Paenibacillaceae.Brevibacillus	5.75117228			-
Bacteria.Chloroflexi.Chloroflexia.Chloroflexales	6.308405095			-
Bacteria.Proteobacteria.Betaproteobacteria.Rhodocyclales.Rhodocyclaceae	4.562779931			-
Bacteria.Firmicutes.Erysipelotrichia.Erysipelotrichales.Erysipelotrichaceae.Erysipelothrix	5.277905991	NF	4.966620279	0.027323722
Bacteria.Proteobacteria.Deltaproteobacteria.Myxococcales.Nannocystaceae	5.63703855	NF	5.147777773	0.027323722
Bacteria.Proteobacteria.Betaproteobacteria.Burkholderiales	3.774471411			-
Bacteria.Actinobacteria.Actinobacteria.Micrococcales.Promicromonosporaceae	5.265429087			-
Bacteria.Actinobacteria.Actinobacteria.Corynebacteriales.Dietziaceae	5.72168855	F_3	5.420714427	0.027323722
Bacteria.Bacteroidetes.Sphingobacteriia.Sphingobacteriales.Chitinophagaceae.Niastella	5.634162562			-
Bacteria.Actinobacteria.Actinobacteria.Solirubrobacterales.Patulibacteraceae	5.369206282			-
Bacteria.Proteobacteria.Betaproteobacteria.Rhodocyclales.Rhodocyclaceae.Azospira	4.427439767	NF	4.339764285	0.022109128
Bacteria.Proteobacteria.Alphaproteobacteria.Rhodobacterales.Rhodobacteraceae	5.53793556	NF	5.204199517	0.03899022
Bacteria.Actinobacteria.Actinobacteria.Corynebacteriales.Corynebacteriaceae.Corynebacterium_1	6.955421757	F_3	6.621299692	0.027323722
Bacteria.Proteobacteria.Alphaproteobacteria.Rickettsiales	5.076931656			-
Bacteria.Bacteroidetes.Sphingobacteriia.Sphingobacteriales.Sphingobacteriaceae.Olivibacter	5.551714695			-
Bacteria.Proteobacteria.Alphaproteobacteria.Rhizobiales.Phyllobacteriaceae.Aminobacter	5.920749715			-
Bacteria.Proteobacteria.Deltaproteobacteria.Myxococcales.Amb_16S_1034	4.124938571			-
Bacteria.Proteobacteria.Betaproteobacteria.Hydrogenophilales	4.467113165			-
Bacteria.Verrucomicrobia.Verrucomicrobiae	6.279076567			-
Bacteria.Actinobacteria.Actinobacteria.Corynebacteriales.Nocardiaceae	5.680413879			-
Bacteria.Firmicutes.Bacilli.Bacillales.Paenibacillaceae.Thermobacillus	3.647598157			-
Bacteria.Chloroflexi.Thermomicrobia.Sphaerobacterales.Sphaerobacteraceae.Sphaerobacter	3.497353564			-
Bacteria.Bacteroidetes.Sphingobacteriia.Sphingobacteriales.Saprospiraceae	6.172889296	NF	5.865525277	0.027323722

Bacteria.Proteobacteria.Alphaproteobacteria.Rhodobacterales.Rhodobacteraceae.Rhodobacter	5.080012204			-
Bacteria.Chloroflexi.TK10	6.248777423			-
Bacteria.Actinobacteria.Actinobacteria.Corynebacteriales.Mycobacteriaceae	6.08724414	F_3	5.668059024	0.027323722
Bacteria.Bacteroidetes.Flavobacteriia.Flavobacteriales.Flavobacteriaceae.Myroides	5.033168472			-
Bacteria.Chlorobi	5.991195812	NF	5.471302963	0.027323722
Bacteria.Firmicutes.Bacilli.Bacillales.Bacillaceae.Lentibacillus	5.191885164	F_3	4.893845085	0.045938131
Bacteria.Planctomycetes.Phycisphaerae.Phycisphaerales.Phycisphaeraceae	5.756798329			-
Bacteria.Bacteroidetes.Bacteroidia	5.797201593			-
Bacteria.Chlamydiae.Chlamydiae.Chlamydiales.Simkaniaceae.Candidatus_Fritschea	3.869446796			-
Bacteria.Actinobacteria.Actinobacteria.Micrococcales.Microbacteriaceae	6.598135971			-
Bacteria.Firmicutes.Bacilli.Bacillales.Thermoactinomycetaceae	4.942302901			-
Bacteria.Actinobacteria.Actinobacteria.Acidimicrobiales.Acidimicrobiales_Incertae_Sedis.Candidatus_Microthrix	4.791724249			-
Bacteria.Proteobacteria.Gammaproteobacteria.Oceanospirillales.Alcanivoracaceae	4.836646109			-
Bacteria.Actinobacteria.Actinobacteria.Pseudonocardiales.Pseudonocardiaceae.Saccharomonospora	3.869446796			-
Bacteria.Chloroflexi.Gitt_GS_136	6.736544052	F_3	6.389104921	0.027323722
Bacteria.Proteobacteria.Betaproteobacteria.Burkholderiales.Comamonadaceae.Schlegelella	4.402447142			-
Bacteria.Firmicutes.Bacilli.Bacillales.Planococcaceae.Planomicrobium	4.444547137	NF	4.4117656	0.022109128
Bacteria.Actinobacteria.Actinobacteria.Solirubrobacterales.Conexibacteraceae	5.356501503			-
Bacteria.Verrucomicrobia.Spartobacteria.Chthoniobacterales.FukuN18_freshwater_group	5.860813193			-
Bacteria.Actinobacteria.Actinobacteria.Propionibacteriales.Propionibacteriaceae.Haloactinopolyspora	4.486860167			-
Bacteria.Proteobacteria.Betaproteobacteria.Neisseriales	4.900191108			-
Bacteria.Proteobacteria.Gammaproteobacteria.Xanthomonadales.Xanthomonadaceae.Pseudof	4.1255802			-

ulvimonas				
Bacteria.Proteobacteria.Gammaproteobacteria.Chromatiales.Ectothiorhodospiraceae	5.259053714			-
Bacteria.Proteobacteria.Alphaproteobacteria.Rickettsiales.Rickettsiales_Incertae_Sedis.Candidatus_Odyssella	5.056546889			-
Bacteria.Deinococcus_Thermus.Deinococci.Deinococcales	5.827683817			-
Bacteria.Proteobacteria.Betaproteobacteria.Burkholderiales.Alcaligenaceae	5.281846797			-
Bacteria.Proteobacteria.Gammaproteobacteria.Enterobacteriales.Enterobacteriaceae.Raoultella	5.30956288			-
Bacteria.Proteobacteria.Epsilonproteobacteria	4.892283427			-
Bacteria.Bacteroidetes.Sphingobacteriia.Sphingobacteriales.NS11_12_marine_group	5.832903006	NF	5.449462033	0.027323722
Bacteria.Proteobacteria.Alphaproteobacteria.Rhizobiales.Hyphomicrobiaceae	5.500602351			-
Bacteria.Proteobacteria.Gammaproteobacteria.NKB5	6.451343707	F_3	6.093446649	0.027323722
Bacteria.Proteobacteria.Gammaproteobacteria.Enterobacteriales.Enterobacteriaceae.Escherichia_Shigella	4.351707795			-
Bacteria.Spirochaetae.Spirochaetes.Spirochaetales.Spirochaetaceae.Spirochaeta_2	4.889474336			-
Bacteria.Firmicutes.Clostridia.Clostridiales.Family_XI.Gottschalkia	5.266674572			-
Bacteria.Gemmatimonadetes.Gemmatimonadetes.AT425_EubC11_terrestrial_group	6.004135499	NF	5.517938791	0.027323722
Bacteria.Nitrospirae	6.456736143	NF	6.080925629	0.03899022
Bacteria.Actinobacteria.Actinobacteria.Streptosporangiales.Streptosporangiaceae	5.300743118			-
Bacteria.Verrucomicrobia.Spartobacteria.Chthoniobacterales.Xiphinematobacteraceae.Candidatus_Xiphinematobacter	5.189292618			-
Bacteria.Planctomycetes	6.815675607	F_3	6.236822987	0.027323722
Bacteria.Proteobacteria.Betaproteobacteria.Burkholderiales.Alcaligenaceae.Paenalcaligenes	4.716880236			-
Bacteria.Proteobacteria.Alphaproteobacteria.Sphingomonadales.Erythrobacteraceae	5.892698095			-
Bacteria.Bacteroidetes.Cytophagia.Cytophagales.Cytophagaceae.Emticicia	4.972110516			-

Bacteria.Firmicutes.Clostridia.Clostridiales.Clostridiaceae_1.Clostridium_sensu_stricto_1	5.141507091			-
Bacteria.Proteobacteria.Alphaproteobacteria.Rickettsiales.Rickettsiales_Incertae_Sedis	5.194701892			-
Bacteria.Firmicutes.Clostridia.Clostridiales.Clostridiaceae_1.Clostridium_sensu_stricto_3	4.030532262			-
Bacteria.Actinobacteria.Actinobacteria.Catenulisporales	4.170476792			-
Bacteria.Firmicutes.Bacilli.Bacillales.Sporolactobacillaceae.Sporolactobacillus	4.08017419			-
Bacteria.Proteobacteria.Alphaproteobacteria.Rhodospirillales.Rhodospirillaceae.Telmatospirillum	4.115576002			-
Bacteria.Firmicutes.Clostridia.Clostridiales.Clostridiaceae_1.Clostridium_sensu_stricto_8	4.183613712			-
Bacteria.Chlamydiae.Chlamydiae	6.71018454	F_3	6.325792756	0.027323722
Bacteria.Proteobacteria.Deltaproteobacteria.Myxococcales.Myxococcaceae.Myxococcus	4.765888742			-
Bacteria.Actinobacteria.Actinobacteria.Glycomycetales.Glycomycetaceae	4.463861735			-
Bacteria.Planctomycetes.BD7_11	4.471726981			-
Bacteria.Chloroflexi.Anaerolineae.Anaerolineales	7.240987103	NF	6.767081324	0.03899022
Bacteria.Bacteroidetes.Sphingobacteriia.Sphingobacteriales.Chitinophagaceae.Flavisolibacter	6.676959613			-
Bacteria.Chlamydiae.Chlamydiae.Chlamydiales.Simkaniaceae	4.73101252			-
Bacteria.Chloroflexi.Anaerolineae.Anaerolineales.Anaerolineaceae	7.240507634	NF	6.779966499	0.03899022
Bacteria.Actinobacteria.Actinobacteria.Solirubrobacterales.Elev_16S_1332	4.36890482			-
Bacteria.Firmicutes.Bacilli.Lactobacillales.Lactobacillaceae	5.735115578			-
Bacteria.Chloroflexi	5.40464355			-
Bacteria.Cyanobacteria.Cyanobacteria.SubsectionIV.FamilyI	4.778567941			-
Bacteria.Proteobacteria.Gammaproteobacteria.Xanthomonadales.Xanthomonadaceae.Lysobacter	6.347665512			-
Bacteria.Actinobacteria.Actinobacteria.Corynebacteriales.Segniliparaceae	3.948628152			-
Bacteria.Proteobacteria.Betaproteobacteria.Burkholderiales.Oxalobacteraceae.Noviherbaspirillum	4.254797724			-

Bacteria.Actinobacteria.Actinobacteria.Solirubrobacterales.0319_6M6	5.060996794			-
Bacteria.Bacteroidetes.Sphingobacteriia.Sphingobacteriales.Sphingobacteriaceae.Arcticibacter	5.189292886	F_3	4.861937368	0.03899022
Bacteria.Proteobacteria.Alphaproteobacteria.Rickettsiales.Anaplasmataceae.Candidatus_Xenohaliotis	5.78402707	F_1	5.494319283	0.034735259
Bacteria.Proteobacteria.Alphaproteobacteria.Rhizobiales.Rhodobiaceae	5.526123812	NF	5.186731691	0.027323722
Bacteria.Chloroflexi.Ktedonobacteria.JG30_KF_AS9	3.804662623			-
Bacteria.Proteobacteria.Deltaproteobacteria.Oligoflexales.Oligoflexaceae.Oligoflexus	5.043675127			-
Bacteria.Chloroflexi.Chloroflexia.Chloroflexales.Chloroflexaceae.Chloronema	3.966576015			-
Bacteria.Deinococcus_Thermus	5.827683817			-
Bacteria.Firmicutes.Clostridia	4.251811825			-
Bacteria.Firmicutes.Bacilli.Lactobacillales.Leuconostocaceae	4.496007166	F_3	4.654007928	0.045938131
Bacteria.Bacteroidetes.Flavobacteriia.Flavobacteriales.Flavobacteriaceae.Ornithobacterium	4.115576002			-
Bacteria.Planctomycetes.Planctomycetacia	6.522703503	F_3	6.149325406	0.027323722
Bacteria.Gracilibacteria	5.166367689			-
Bacteria.Acidobacteria.Acidobacteria.Subgroup_4.DS_100	5.182213688			-
Bacteria.Fibrobacteres.Fibrobacteria.Fibrobacterales.Fibrobacteraceae.possible_genus_04	5.237421661	F_3	4.950861174	0.027323722
Bacteria.Actinobacteria.Actinobacteria.Pseudonocardiales	6.070222861	F_3	5.722260273	0.027323722
Bacteria.Omnitrophica	5.232740697	F_3	4.983268513	0.03899022
Bacteria.Chloroflexi.SAR202_clade	4.543789119	F_1	4.328910243	0.049647804
Bacteria.SHA_109	5.531912312			-
Bacteria.Actinobacteria.Actinobacteria.Acidimicrobiales.Iamiaceae	5.680546504			-
Bacteria.Proteobacteria.Alphaproteobacteria.Sphingomonadales.Sphingomonadaceae.Sphingosinicella	5.562182765			-
Bacteria.Proteobacteria.Betaproteobacteria.Methylophilales.Methylophilaceae.Methylophilus	4.440186476	NF	4.54311569	0.034735259
Bacteria.Actinobacteria.Actinobacteria.Frankiales.Acidothermaceae.Acidothermus	3.876625464			-

Bacteria.Bacteroidetes.Cytophagia.Cytophagales.Cyclobacteriaceae.Algoriphagus	5.594487935			-
Bacteria.Actinobacteria.Actinobacteria.Rubrobacterales	4.709563093	NF	4.576198529	0.037941542
Bacteria.Firmicutes.Bacilli.Lactobacillales.Carnobacteriaceae.Alkalibacterium	4.057437303			-
Bacteria.Firmicutes.Bacilli.Bacillales.Planococcaceae.Sporosarcina	5.82682873			-
Bacteria.Bacteroidetes.Flavobacteriia.Flavobacteriales.Flavobacteriaceae.Sinomicrobium	3.647598157			-
Bacteria.Cyanobacteria.Cyanobacteria.SubsectionI	4.482418354			-
Bacteria.Saccharibacteria	7.631545768	F_3	7.216205482	0.03899022
Bacteria.Proteobacteria.Gammaproteobacteria.Legionellales.Legionellaceae.Legionella	5.87582646	F_3	5.530982553	0.03899022
Bacteria.Bacteroidetes.Cytophagia.Cytophagales.Cyclobacteriaceae	4.840477375			-
Bacteria.Proteobacteria.Gammaproteobacteria.Cellvibrionales.Porticoccaceae.C1_B045	4.826403365			-
Bacteria.Bacteroidetes.Flavobacteriia.Flavobacteriales.Cryomorphaceae.Owenweeksia	4.200659254			-
Bacteria.Bacteroidetes.Sphingobacteriia.Sphingobacteriales.Saprospiraceae.Phaeodactylibacter	4.17765546			-
Bacteria.Fibrobacteres.Fibrobacteria	5.601197577			-
Bacteria.Firmicutes.Clostridia.Clostridiales.Lachnospiraceae.Epulopiscium	3.860303446			-
Bacteria.Proteobacteria.Deltaproteobacteria.Desulfobacterales.Nitrospinaceae	4.510199774			-
Bacteria.Actinobacteria.Actinobacteria.Solirubrobacterales.480_2	5.706261538			-
Bacteria.Proteobacteria.Deltaproteobacteria.Desulfobacterales.Nitrospinaceae.Candidatus_Entotheonella	4.448893249	NF	4.378073582	0.022109128
Bacteria.Bacteroidetes.Sphingobacteriia.Sphingobacteriales.Chitinophagaceae.Parafilimonas	5.33552706	F_3	5.062186653	0.0265095
Bacteria.Firmicutes.Bacilli.Lactobacillales.Enterococcaceae.Enterococcus	5.707840379	F_3	5.342056215	0.027323722
Bacteria.Firmicutes.Bacilli.Bacillales.Paenibacillaceae.Cohnella	5.499876118			-
Bacteria.Verrucomicrobia.Opitutae.Opitutae_vadinHA64	5.693004619			-
Bacteria.Deinococcus_Thermus.Deinococci.Deinococcales.Trueperaceae	5.827683817			-
Bacteria.Firmicutes.Bacilli.Bacillales.Paenibacillaceae	5.013015999			-

Bacteria.Actinobacteria.Actinobacteria.Micrococcales.Brevibacteriaceae.Brevibacterium	6.460233439	F_3	6.138840013	0.027323722
Bacteria.Proteobacteria.Gammaproteobacteria.KI89A_clade	4.687524681			-
Bacteria.Proteobacteria.Alphaproteobacteria.Rhizobiales.Rhizobiales_Incertae_Sedis.Bauldia	5.799814404			-
Bacteria.Firmicutes.Bacilli.Bacillales.Paenibacillaceae.Oxalophagus	6.2059956			-
Bacteria.Proteobacteria.Gammaproteobacteria.Xanthomonadales.JTB255_marine_benthic_group	4.375868103			-
Bacteria.Verrucomicrobia.Spartobacteria.Chthoniobacterales.Xiphinematobacteraceae	5.189292618			-
Bacteria.Firmicutes.Bacilli.Bacillales.Planococcaceae.Psychrobacillus	5.707615194			-
Bacteria.Verrucomicrobia.Verrucomicrobiae.Verrucomicrobiales.Verrucomicrobiaceae.Haleferula	5.533938741			-
Bacteria.Actinobacteria.Actinobacteria.Streptosporangiales.Nocardiopsaceae.Nocardiopsis	5.184115452	F_1	4.860718081	0.049647804
Bacteria.Proteobacteria.Gammaproteobacteria.Oceanospirillales.Alcanivoracaceae.Alcanivorax	4.836646109			-
Bacteria.Proteobacteria.Betaproteobacteria.SC_I_84	6.197535797			-
Bacteria.Bacteroidetes.Flavobacteriia.Flavobacteriales.Flavobacteriaceae.Subsaxibacter	4.764931436			-
Bacteria.Firmicutes.Bacilli.Lactobacillales.Leuconostocaceae.Leuconostoc	4.496007166	F_3	4.594238981	0.045938131
Bacteria.Actinobacteria.Actinobacteria.Streptosporangiales.Thermomonosporaceae	5.969972718	F_3	5.638280489	0.027323722
Bacteria.Proteobacteria.Gammaproteobacteria.Alteromonadales.Idiomarinaceae.Idiomarina	4.597004374			-
Bacteria.Proteobacteria.Gammaproteobacteria.Thiotrichales.Thiotrichaceae	5.156907683	F_3	4.900888215	0.0265095
Bacteria.Proteobacteria.Alphaproteobacteria.Rhizobiales.DUNssu371	5.087150147			-
Bacteria.Actinobacteria.Actinobacteria.Pseudonocardiales.Pseudonocardiaceae.Pseudonocardia	5.797531447	F_3	5.451285578	0.03899022
Bacteria.Proteobacteria.Gammaproteobacteria.Thiotrichales.Piscirickettsiaceae	4.747818359			-
Bacteria.Verrucomicrobia.Verrucomicrobiae.Verrucomicrobiales	6.279076567			-
Bacteria.Proteobacteria.Deltaproteobacteria.Desulfurellales.Desulfurellaceae	3.647598157			-

Bacteria.Proteobacteria.Alphaproteobacteria.Rhodospirillales.KCM_B_15	5.297279969			-
Bacteria.Firmicutes.Clostridia.Clostridiales.Peptostreptococcaceae.Peptoclostridium	4.524524867			-
Bacteria.Proteobacteria.Gammaproteobacteria.Aeromonadales.Aeromonadaceae	5.542745791			-
Bacteria.Acidobacteria.Acidobacteria.Acidobacteriales.Acidobacteriaceae_Subgroup_1	5.641437412			-
Bacteria.Proteobacteria.Betaproteobacteria.Burkholderiales.Oxalobacteraceae.Massilia	6.142976215	NF	5.78564578	0.03899022
Bacteria.Bacteroidetes.Sphingobacteriia.Sphingobacteriales	4.989507069			-
Bacteria.Actinobacteria	7.680839075			-
Bacteria.Proteobacteria.Betaproteobacteria.Neisseriales.Neisseriaceae.Vogesella	3.5098222			-
Bacteria.Firmicutes.Bacilli.Bacillales.Sporolactobacillaceae	3.723318839			-
Bacteria.Actinobacteria.Actinobacteria.Solirubrobacterales.FFCH13075	3.876625464			-
Bacteria.Proteobacteria.Alphaproteobacteria.Rhizobiales.Methylobacteriaceae.Microvirga	5.957817133			-
Bacteria.Proteobacteria.Alphaproteobacteria.Rhodospirillales.Rhodospirillales_Incertae_Sedis	6.407976911			-
Bacteria.Acidobacteria.Acidobacteria.Subgroup_9	4.192702726			-
Bacteria.Acidobacteria.Acidobacteria.Subgroup_17	5.406750559			-
Bacteria.Proteobacteria.Alphaproteobacteria.Sphingomonadales.Sphingomonadaceae.Sphingobium	5.553644456	F_1	5.194365372	0.027323722
Bacteria.Chloroflexi.Ardenticatenia.Ardenticatenales	5.332874674			-
Bacteria.Actinobacteria.Actinobacteria.Bifidobacteriales.Bifidobacteriaceae	4.69780032			-
Bacteria.Proteobacteria.Betaproteobacteria.Burkholderiales.Comamonadaceae.Hydrogenophaga	5.203185376	F_3	4.921075168	0.027323722
Bacteria.Proteobacteria.Betaproteobacteria.Methylophilales.Methylophilaceae.Methylobacillus	5.491966545	NF	5.112574971	0.027323722
Bacteria.Proteobacteria.Alphaproteobacteria.Rhizobiales.Xanthobacteraceae.Variibacter	6.394525809			-
Bacteria.Gemmatimonadetes.Gemmatimonadetes	6.349050965			-
Bacteria.Proteobacteria.Alphaproteobacteria.Rhodospirillales.MSB_1E8	4.690814326			-
Bacteria.Firmicutes.Bacilli.Bacillales.Planococcaceae.Domibacillus	5.430256271			-

Bacteria.Proteobacteria.Deltaproteobacteria.Myxococcales.Nannocystaceae.Pseudenhygromyxa	5.630564963			-
Bacteria.Acidobacteria.Acidobacteria.Subgroup_5	5.463874612			-
Bacteria.Proteobacteria.Alphaproteobacteria.Rhodospirillales.I_10	6.054061889	NF	5.548092116	0.03899022
Bacteria.Chloroflexi.Caldilineae.Caldilineales	5.976148521			-
Bacteria.Actinobacteria.Actinobacteria.Glycomycetales.Glycomycetaceae.Glycomyces	4.463861735			-
Bacteria.Acidobacteria.Acidobacteria.Subgroup_6	7.291378429			-
Bacteria.Verrucomicrobia.Opitutae.101_154	3.774471411			-
Bacteria.Proteobacteria.Betaproteobacteria.Burkholderiales.Alcaligenaceae.Pigmentiphaga	4.050677799			-
Bacteria.Proteobacteria.Alphaproteobacteria.Rickettsiales.Holosporaceae	6.07889393	F_3	5.666487593	0.027323722
Bacteria.WD272	3.774471411			-
Bacteria.Actinobacteria.Actinobacteria.Pseudonocardiales.Pseudonocardiaceae.Crossiella	3.869446796			-
Bacteria.Proteobacteria.Alphaproteobacteria.Rickettsiales.Rickettsiaceae.Rickettsia	4.779469599	F_3	4.639088696	0.049647804
Bacteria.Bacteroidetes.Sphingobacteriia	7.548208577	F_3	7.046647589	0.027323722
Bacteria.Proteobacteria.Betaproteobacteria.Burkholderiales.Burkholderiaceae.Ralstonia	4.867125628			-
Bacteria.Actinobacteria.Actinobacteria.Corynebacteriales.Mycobacteriaceae.Mycobacterium	6.08724414	F_3	5.700476812	0.027323722
Bacteria.Chloroflexi.Anaerolineae	7.240987103	NF	6.788258412	0.03899022
Bacteria.Bacteroidetes.Bacteroidia.Bacteroidales.Porphyromonadaceae	4.490263457	NF	4.78213297	0.022109128
Bacteria.Chlorobi.Ignavibacteria.Ignavibacteriales	5.575625658	F_1	5.295122122	0.049647804
Bacteria.Acidobacteria.Acidobacteria.Subgroup_15	4.684687758	F_1	4.471636594	0.034863256
Bacteria.Acidobacteria.Acidobacteria.Subgroup_12	3.61956934			-
Bacteria.Acidobacteria.Acidobacteria.Subgroup_13	3.61956934			-
Bacteria.Acidobacteria.Acidobacteria.Subgroup_10	6.069865932	NF	5.624629321	0.03899022
Bacteria.Acidobacteria.Acidobacteria.Subgroup_11	4.519339811			-
Bacteria.Acidobacteria.Acidobacteria.Subgroup_4	7.082524182	F_3	6.68735721	0.03899022

Bacteria.Cyanobacteria.Cyanobacteria	6.041497109			-
Bacteria.Proteobacteria.Deltaproteobacteria.Myxococcales.Cystobacteraceae.Anaeromyxobacter	4.057437303			-
Bacteria.Acidobacteria.Acidobacteria.Subgroup_7	6.496673443			-
Bacteria	6			-
Bacteria.Acidobacteria.Acidobacteria.Subgroup_3	6.607415315			-
